# Reliable Modeling of Anharmonic Spectra Line-Shapes from VPT2 and Hybrid QM Models: IR Spectrum of Uracil as a Test Case

**DOI:** 10.1021/acs.jpca.5c02226

**Published:** 2025-06-25

**Authors:** Ruiqin Xu, Qin Yang, Julien Bloino, Malgorzata Biczysko

**Affiliations:** † Department of Physics, College of Sciences, Shanghai University, 99 Shangda Road, Shanghai 200444, China; ‡ Institute of Organic Chemistry and Biochemistry, Czech Academy of Science, Flemingovo náměstí 2, 16610 Prague, Czech Republic; ¶ Classe di Scienze, 19004 Scuola Normale Superiore, Piazza dei Cavalieri 7, 56126 Pisa, Italy; § Faculty of Chemistry, 49572 University of Wroclaw, F. Joliot-Curie 14, 50-383 Wroclaw, Poland

## Abstract

Hybrid methods combining different levels of electronic structure quantum mechanical computations with vibrational perturbation theory have been increasingly used in anharmonic simulations of vibrational spectra to achieve accurate results with containable computational costs. However, energy has often been the main focus of these studies, so precision in predicting intensities was systematically overlooked. This situation is largely due to two aspects stemming from theory and experiment. Theoretically, implementations are fewer, and intensity-specific resonance analysis methods were not available until very recently and are still lacking extensive testing. Experimentally, high-resolution vibrational spectra of suitable molecular systems, which could really show the effects of different hybrid schemes on the vibrational intensities, remain scarce. A good candidate in this regard is uracil. Having been extensively studied experimentally, its IR spectrum is well-known over a wide range and at high definition. The patterns displayed by the band shapes represent an excellent challenge to validate and tune our recently developed automated tool to identify intensity-specific resonances. In this work, we compare the newly simulated spectra with state-of-the-art experimental data and propose an extensive analysis over a wide range covering 300 to 6200 cm^–1^, including 3-quanta transitions. These will provide valuable guides and references for further measurements in the mid-infrared (MIR) and near-infrared (NIR) regions, which have not been reported until now. The methods and protocols applied in this article can also be used for other molecules with complex resonance patterns.

## Graphical abstract

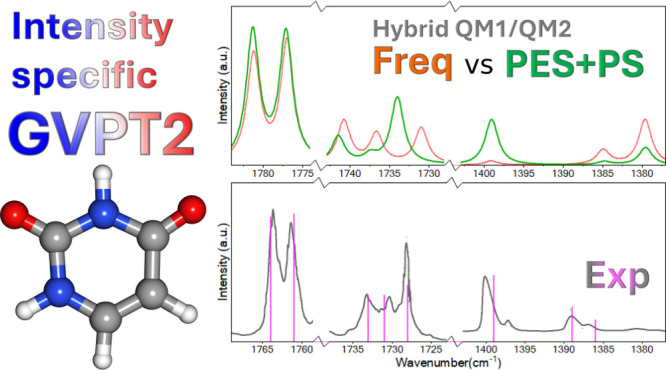

## Introduction

Vibrational spectroscopy is a powerful means for investigating the structural and dynamical properties of molecular systems, for which interpretation and analysis are nowadays routinely assisted by computations.^1,2^ These can range from the simplest harmonic ones that yield only fundamental transitions and at best allow only a rough description of the most significant features of the spectra to highly accurate anharmonic simulations accessible only to very small molecules.^3–6^ The starting point for the simulation of any vibrational spectrum is the definition of the vibrational levels, which govern the band positions. From a computational perspective, it depends on the description of the potential energy surface (PES), which can be done with a wide range of electronic structure quantum mechanical (ESQM) methods. The situation is different for the band intensities. Depending on the spectroscopy of interest, different properties can be involved, which may or may not be available for a given electronic structure calculation method. In turn, this affects the achievable level of accuracy for the prediction of a given type of spectrum. In that context, infrared (IR) absorption is a good target. Indeed, analytic forms of the electric dipole and often its first derivatives as well, necessary to predict the intensities at the harmonic level, have been proposed and implemented for a wide range of ESQM methods. Thus, it is possible to extensively test computational protocols and establish precisely the achievable accuracy for different combinations of the electronic and nuclear levels of theory. This represents a crucial step in the exploration of other vibrational spectroscopic techniques, such as Raman scattering,^7^ vibrational circular dichroism (VCD)^8,9^ or Raman optical activity (ROA).^10^ Indeed, great advances have been achieved in the derivation of appropriate properties thanks to developments within the response function theory,^11–16^ and constant efforts are made to extend the computational support of vibrational spectroscopies.^17^ However, in practice, many properties and the necessary derivatives are still available only at the density functional theory (DFT) level,^1,9^ excluding double hybrid DFT models.

In this work, we continue our efforts in the development and validation of protocols for the simulation of vibrational spectral line shapes beyond the harmonic approximation, considering both mechanical and electrical anharmonic corrections. The main focus here is on the relative band intensities, including strong nonfundamental transitions,^18^ which are often related to resonances. These effects are important for all spectroscopies, posing a further complication in anharmonic spectral simulations due to the sensitivity of band intensities to even subtle resonance effects.^19^

The PES and property surface (PS) are the primary ingredients in the simulation of vibrational spectra, and the quality of their description directly affects the accuracy of the result. Besides the level of theory used for the electronic structure calculations, an important factor is the representation of nuclear motions, especially vibrations. Achieving spectroscopic accuracy requires going beyond the harmonic-oscillator approximation, which can be done with vibrational perturbative^2,20^ or variational approaches.^21,22^

For IR spectra, the most refined ESQM methods, such as coupled cluster (CC), including CCSD­(T),^23–26^ or approaches based on configuration interaction (CI), in conjunction with essentially converged basis sets, providing highly accurate and reliable results, can be applied. But, in practice, anharmonic PESs and PSs can only be computed at these levels for very small molecular systems. There have been significant efforts to extend CC-based methods to larger systems, employing composite schemes,^27–30^ as well as “cheaper” alternatives, where some contributions are obtained from lower levels of theory, usually second-order Mo̷ller Plesset (MP2) or double-hybrid DFT (DH-DFT).^31,32^ However, such schemes are generally designed to improve the energies and structures. They are not suitable or have rarely been tested for properties.^33^

Comparatively, density functionals represent a very cost-effective alternative, capable of tackling bigger molecules and, more generally, larger problems,^34^ such as the computation of anharmonic constants. For this reason, they have often been preferred as the reference electronic structure calculation method to study the importance of anharmonic effects and design computational protocols for accurate simulations of vibrational spectra.^7,35–40^ Over the years, it has been confirmed that a very effective strategy is to employ hybrid approaches in which a higher level of theory is employed to compute the harmonic energies and properties, and a cheaper one for the anharmonic terms.^2,40–43^ Hybrid force fields combining double hybrid functionals or even highly accurate composite schemes based on Coupled Cluster theory for the harmonic part, with less expensive DFT functionals for the anharmonic part (DH-DFT/DFT and CC/DFT, respectively) have been applied for several molecular systems showing significantly improved accuracy of wavenumbers over pure DFT.^33,44–48^

However, hybrid approaches combining two quantum mechanical methods in a QM1/QM2 scheme have not yet been extensively tested regarding the intensities and the overall spectral profile, in particular considering also the intense nonfundamental transitions. The first challenge now is to establish a comprehensive computational protocol based on such a hybridization technique, which is suitable to describe properties and observables beyond the energies.

In this work, we will employ vibrational perturbation theory at the second order (VPT2), with PESs and PSs expanded up to the fourth and third orders, respectively.^20,49–55^ A well-known issue with employing VPT2 is its propensity to diverge in the presence of singularities caused by small energy differences between states at the harmonic level, leading to unrealistic energies and intensities. The strategy for recovering the correct values is now consolidated and consists of two steps. First, resonant terms are identified and removed, and second, the missing correction is recovered through a variational step. The first part is the most challenging, as the definition of resonances is not univocal. As a result, several criteria to identify resonances have been proposed in the literature.^40,56–58^ Most of these were designed and tested on small systems. However, scaling up to larger systems is not trivial as resonances often overlap, complicating the process of identifying the precise source of errors. Fine-tuning the criteria and associated thresholds to achieve proper identification of resonances while avoiding over- or undercorrection proves to be challenging. Recently, some of us developed and implemented a new automated protocol, which takes into account the specific definitions of the transition energies and intensities to identify systematically resonances.^19^ This approach is used and extensively tested in the context of hybrid QM1/QM2 models in this work, within the generalized VPT2 model (GVPT2).^7,53,59^

As a test case, we chose the IR spectrum of uracil, one of the four RNA nucleobases, whose diketo-tautomeric form is known to be the most stable^60,61^ (see Figure 1). An interesting feature of uracil’s IR spectra is its multiple Fermi resonances in the mid-IR (MIR) region.^62–65^ Of particular relevance is the spectral range 1600–1800 cm^–1^, where several overtones and combination transitions contribute considerable intensities. They are even comparable to the fundamentals related to carbonyl stretchings (ν_C=O_), resulting in congestion of this part of the spectrum and a complex experimental line shape to interpret. Over the past decades, various spectroscopic experiments and theoretical computations on uracil have been carried out. For instance, the IR and Raman spectra of uracil have been measured in the gas phase^66,67^ dissolved,^68,69^ as polycrystalline,^70–72^ and isolated in low temperature matrices.^62,63,73–79^ The best resolved spectral line-shape to date has been reported by Barnes et al.^62^ and Ivanov et al.^63^ These experiments have been followed by several theoretical studies carried out to predict the fundamental and nonfundamental bands of isolated uracil beyond the harmonic approximation.^33,44,64,65,80–87^ Thanks to the determination of harmonic wavenumbers through the CCSD­(T)-based composite scheme,^33^ it was possible to set up CC/DFT^7,33,44^ or CC/MP2 ^64^ hybrid methods, yielding very accurate wavenumbers for fundamental and nonfundamental transitions. In previous works by some of us,^7,33,44^ the identification of resonances was performed in different ways: automatic procedures based on pure DFT force constants and hybrid CC/DFT force field, completed by manual definitions based on the contact-transformation VPT2 analysis by Ten et al.,^82^ with all schemes providing very similar results, reaching a mean absolute error (MAE) of 11 cm^–1^ over all assigned fundamental and nonfundamental transitions. The following studies have shown that GVPT2 predicts a qualitatively correct pattern in the 1600–1800 cm^–1^ range.^65^ Studies by Krasnoshchekov’s group^64,82^ also reported anharmonic resonance polyads, as well as a more detailed analysis of the spectral line shape employing the canonical (CVPT2) algorithm on top of MP2 calculations. They considered together available experimental infrared and Raman data, achieving the most reliable assignments of uracil vibrations to date.^64^ This work will also be used as a reference in our analysis of uracil spectra with a recently introduced effective and automated processing of resonances.^19^

**Figure 1:**
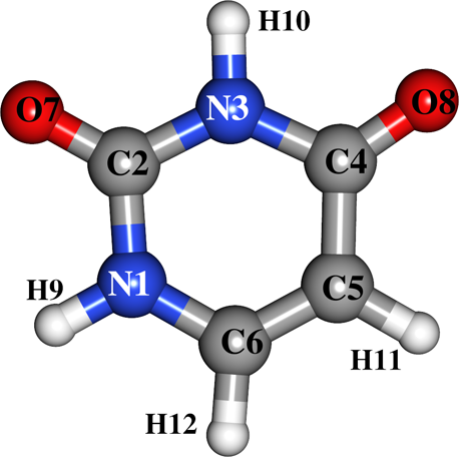
Molecular structure of uracil with atomic labels based on previous works.^31,33^

The aim of this work is thus dual. First, we tested the QM1/QM2 schemes for the simulation of anharmonic intensities. Then, we apply the most reliable methods to derive reference PES and PS data and check the robustness of GVPT2 with a recently introduced automated protocol^19^ to identify and correct all relevant resonances. This will be used to simulate the IR spectral line-shape, including patterns affected by strong Fermi resonances.

The paper is organized as follows. In the next section, the necessary theoretical aspects are summarized and followed by the computational details relevant for DFT and VPT2 computations. The DFT results for the equilibrium parameters are then compared with the best available estimates to select the DFT methods to be applied in spectral studies. That group is then used to build hybrid PESs and PSs, and their quality is tested over a selection of well-defined bands. Finally, the IR spectrum is analyzed in detail, focusing on the assignments of all visible fundamental, as well as nonfundamental, bands. The conclusions with guidelines for carrying out similar simulations of fully anharmonic spectra on semirigid molecular systems are provided at the end.

## Computational Details

### DFT Computations

Electronic structure and spectral computations are performed using the double-hybrid density functional revDSD-PBEP86 ^88^ and the hybrid functional B3PW91 ^89^ including Grimme’s D3 dispersion correction with Becke–Johnson (BJ) damping,^90,91^ in conjunction with the calendar basis set jun-cc-pVTZ.^92^ The efficiency of this combination has been validated by several benchmark studies,^40,42^ and it was also used as a reference to validate the method applied here to automatically identify resonances in energies and intensities.^19^

In a first step, the structure of uracil was optimized with very tight convergence criteria, at both revDSD-PBEP86-D3­(BJ)/jun-cc-pVTZ and B3PW91-D3­(BJ)/jun-cc-pVTZ levels. The resulting geometries served as the basis for vibrational harmonic and anharmonic calculations. revDSD-PBEP86-D3­(BJ)/jun-cc-pVTZ computations have also been used for the analysis of the potential energy distribution (PED) employing the GAR2PED^93^ package. The anharmonic PES and PS were generated at the B3PW91-D3­(BJ)/jun-cc-pVTZ level. Namely, cubic and semidiagonal quartic force constants, as well as the second and semidiagonal third derivatives of the electric dipole, were built by numerical differentiations of analytical second derivatives of the energy and first derivatives of the dipole, respectively, as implemented in Gaussian16. The differentiation was done starting from the optimized structure obtained with very tight convergence criteria, using a step along the mass-weighted normal coordinates of $0.01\sqrt{u}Å$ .

### Hybrid Schemes

In this work, since the focus is on the intensities, we have tested different approaches to generate the hybrid potential and property surfaces. The concept of hybrid PESs or PSs refers here to the combination of different levels of electronic structure calculations to represent the order of expansion of a quantity expressed as a Taylor series. Indeed, since the analytic forms of the potential energy *V* or the properties involved in the spectroscopy of interest, here the electric dipole **μ**, are not available, they are typically expanded in Taylor series, up to a target order. Considering a generic quantity θ, an expansion with respect to the dimensionless normal coordinates ***q*** up to the fourth order will be,

$$\theta \left(q\right)=\theta \left(0\right)+\overset{N}{\underset{i=1}{\sum }}\frac{\partial \theta }{\partial q_{i}}q_{i}+\frac{1}{2}\overset{N}{\underset{i,j=1}{\sum }}\frac{\partial^{2}\theta }{\partial q_{i}\partial q_{j}}q_{i}q_{j}+\frac{1}{6}\overset{N}{\underset{i,j,k=1}{\sum }}\frac{\partial^{3}\theta }{\partial q_{i}\partial q_{j}\partial q_{k}}q_{i}q_{j}q_{k}+\frac{1}{24}\overset{N}{\underset{i,j,k,l=1}{\sum }}\frac{\partial^{4}\theta }{\partial q_{i}\partial q_{j}\partial q_{k}\partial q_{l}}q_{i}q_{j}q_{k}q_{l}$$ 1

where **0** represents the equilibrium geometry and the summations are carried out over the *N* normal modes. Let us assume that analytic forms of this quantity are known up to second order, which is typically the case for the potential energy. Then, eq 1 can be divided into two components,

$$\theta =\theta^{H}+\theta^{A}$$ 2

with the superscript “*H*” referring to the harmonic part and “*A*” to the anharmonic extension,

$$\theta {\left(q\right)}^{H}=\theta \left(0\right)+\overset{N}{\underset{i=1}{\sum }}\frac{\partial \theta }{\partial q_{i}}q_{i}+\frac{1}{2}\overset{N}{\underset{i,j=1}{\sum }}\frac{\partial^{2}\theta }{\partial q_{i}\partial q_{j}}q_{i}q_{j}$$ 3

$$\theta {\left(q\right)}^{A}=\frac{1}{6}\overset{N}{\underset{i,j,k=1}{\sum }}\frac{\partial^{3}\theta }{\partial q_{i}\partial q_{j}\partial q_{k}}q_{i}q_{j}q_{k}+\frac{1}{24}\overset{N}{\underset{i,j,k,l=1}{\sum }}\frac{\partial^{4}\theta }{\partial q_{i}\partial q_{j}\partial q_{k}\partial q_{l}}q_{i}q_{j}q_{k}q_{l}$$ 4

It is rather evident that different levels of calculations of θ can be formally combined for the harmonic and anharmonic parts only if the normal coordinates in the two levels are rigorously equal. The affine relation proposed by Duschinsky to connect normal modes from different electronic states for the computation of overlap integrals between vibronic states^94^ can be generalized to any pair of sets of normal coordinates, including those computed at different levels of theory. In this case, the overlap between normal modes, as defined by the Duschinsky matrix (**J**), provides a good measure of their similarity. A value *J*_*ik*_^2^ ≥ 0.9 was used to consider that two normal coordinates were identical between the sets.

An alternative and simpler way to obtain hybrid PESs or PSs is to use higher-level (*H*) normal coordinates to perform numerical differentiation at the lower level and generate the anharmonic constants. However, there are a few drawbacks to this approach. First, the differentiations are potentially done out-of-equilibrium for the lower level, which may introduce numerical noise in the anharmonic constants. Second, the method is not universal. Any combination of ESQMs requires the generation of a new set of anharmonic constants from the beginning, which can be prohibitive when testing different configurations. Since the overlap between the set of normal modes was excellent, the first approach to the hybrid scheme was used.

As a final comment, let us now consider the particularities of the two quantities of interest, the energy and intensity, starting from the former. For the energy, the first derivative (gradient) is null at the equilibrium, and the second derivatives with respect to the normal coordinates correspond to the eigenvalues of the force constant matrix by definition, hence, the vibrational energies. If we ignore the energy at the equilibrium (assuming it is null), and express the potential energies in wavenumbers, the Taylor expansion has the form,

$$V\left(q\right)=\frac{1}{2}\overset{N}{\underset{i=1}{\sum }}{\left[\omega_{i}\right]}^{H}{q_{i}}^{2}+\frac{1}{6}\overset{N}{\underset{i,j,k=1}{\sum }}{\left[\frac{\partial^{3}V}{\partial q_{i}\partial q_{j}\partial q_{k}}\right]}^{A}q_{i}q_{j}q_{k}+\frac{1}{24}\overset{N}{\underset{i,j,k,l=1}{\sum }}{\left[\frac{\partial^{4}V}{\partial q_{i}\partial q_{j}\partial q_{k}\partial q_{l}}\right]}^{A}q_{i}q_{j}q_{k}q_{l}$$ 5

where **ω** is the vector of vibrational energies, here computed at the higher level of theory. In this form, a further simplification seems possible to build the hybrid scheme by simply combining vibrational wavenumbers at the higher level with the anharmonic force field built at a lower level. This shortcut can be convenient when the two sets of normal coordinates are not available, for instance, when using data from the literature. For this reason, such a method will also be considered in the following. However, it should be kept in mind that this would only be valid if the normal coordinates computed at the two levels of theory are perfectly equivalent.

Let us continue this discussion with the case of the intensity. For IR, the spectroscopic observable is related to the molar absorption coefficient, given by

$$\epsilon \left(\omega \right)=\underset{I,F}{\sum }\frac{8\pi^{3}N_{A}\omega }{3hc\ln\left(10\right)}\left|\left\langle \psi_{F}^{v}\right|\mu \right|\psi_{I}^{v}\rangle {|}^{2}\delta \left(\omega -|\omega_{F}-\omega_{I}|\right)$$ 6

where ⟨ψ_*F*_^*v*^|**μ**|ψ_*I*_^*v*^⟩ is the transition moment of the electric dipole between vibrational states |ψ_*I*_^*v*^⟩ and |ψ_*F*_^*v*^⟩. Contrary to the energies, applying a hybrid scheme on the observable (ϵ) is not equivalent to doing so on the expansion of the property in eq 1, because of the cross terms in the product. For this reason, the derivative of the properties for each ESQM must be known to obtain consistent results. In this work, since we assess the quality of hybrid schemes in anharmonic calculations, only combinations at the level of the Taylor expansion of properties were considered.

In summary, three different hybrid QM1/QM2 models have been devised, which match commonly encountered situations. The “Freq” configuration corresponds to typical cases of harmonic QM1 frequencies taken from the literature. These data are used in place of the harmonic wavenumbers from the “low-level” (QM2) calculations, the rest being unchanged. The “PES” version represents an additional refinement, where the high-level normal modes are also known, so the validity of the Taylor expansion for the potential energy (eq 5) can also be checked, and any change in the normal-mode energy order can be automatically corrected. Finally, “PES+PS” represents the most refined model, where the hybrid scheme is consistently applied to the energies and properties. It should be noted that in all three hybrid models, the high-level harmonic wavenumbers are used in transformations and resonance analysis.

### GVPT2 Computations

Regardless of the force field employed, all GVPT2 computations have been performed^7,53,59^ with the recently introduced resonance analysis algorithm, as implemented in the development version of Gaussian and detailed in ref 19. In this version, the GVPT2 approach is a three-step procedure starting from the identification of resonances by combining multiple heuristics. VPT2 energies are then computed, removing all terms associated with the resonances. Next, so-called variational matrices are built for each set of states connected by resonances, called a polyad, starting from the diagonal populated by the resonance-free energies from deperturbed VPT2 (DVPT2). Then, coupling terms between states explicitly in resonances are introduced as off-diagonal elements. The final vibrational energies are obtained by diagonalizing these matrices, while their eigenvectors provide a description of the final GVPT2 states with respect to the DVPT2 states. For further details on the algorithm and its validation, readers are referred to the original work.^19^

It is important to stress that the intensities are not only influenced by Fermi resonances but also heavily impacted by Darling–Dennison resonances. The protocol for the intensities is similar to that of the energy. The DVPT2 transition moments are simply projected onto the GVPT2 states by using the eigenvectors obtained from the energy calculations. As the DVPT2 term was originally used in the context of energy, it can be unclear whether all resonances should be removed independently of their nature or only Fermi resonances. To avoid confusion, we use intensity-extended DVPT2 (IDVPT2) to refer to a more inclusive treatment of resonances by considering all kinds of singularities affecting energies and intensities. Since IDVPT2 and DVPT2 are the same for the energy, IDVPT2 will be used throughout this work to refer to VPT2 calculations, where all resonant terms have been removed.

The criteria used to identify resonances follow ref 19, as summarized here. For Fermi resonances,

$$|\omega_{i}-(\omega_{j}+\omega_{k})|\leq 200\;{\mathrm{cm}}^{-1}$$ 7

$$\frac{f_{ijk}^{4}}{64(1+\delta_{jk}{)}^{2}|\omega_{i}-(\omega_{j}+\omega_{k}){|}^{3}}\geq 1\;{\mathrm{cm}}^{-1}$$ 8

$$\left|\frac{1}{\sqrt{8\left(1+\delta_{ij}\right)}}\frac{f_{ijk}}{\omega_{i}-\left(\omega_{j}+\omega_{k}\right)}\right|\geq 0.1$$ 9

For 1-1 Darling–Dennison resonances,

$$|\omega_{i}-\omega_{j}|\leq 100\;{\mathrm{cm}}^{-1}$$ 10

$$|\langle 1_{i}|H|1_{j}\rangle |\geq 10\;{\mathrm{cm}}^{-1}$$ 11

$$\max\left(|C_{1_{i},1_{j}}^{\left(2\right)}|,|C_{1_{j},1_{i}}^{\left(2\right)}|\right)\geq 1$$ 12

For 1-3 Darling–Dennison resonances,

$$|\omega_{i}-\omega_{j}-\omega_{k}-\omega_{l}|\leq 100\;{\mathrm{cm}}^{-1}$$ 13

$$|\langle 1_{i}|\tilde{H}|1_{j}1_{k}1_{l}\rangle |\geq 10\;{\mathrm{cm}}^{-1}$$ 14

$$\left|C_{1_{i},1_{j},1_{k},1_{l}}^{2}\right|\geq 0.3$$ 15

For 2-2 Darling–Dennison resonances (between overtones or 2-quanta binary combinations),

$$|\omega_{i}+\omega_{j}-(\omega_{k}+\omega_{l})|\leq 100\;{\mathrm{cm}}^{-1}$$ 16

$$|\langle 1_{i}1_{j}|\mathrm{H̃}|1_{k}1_{l}\rangle |\geq 10\;{\mathrm{cm}}^{-1}$$ 17

*f*_*ijk*_ represents the third derivative of the potential energy with respect to the dimensionless normal coordinates, *q*_*i*_, *q*_*j*_, and *q*_*k*_. *H̃* is the contact-transformed Hamiltonian,^95^ and δ is the Kronecker symbol. Further details, along with a description of other available VPT2 schemes concerning both energies and intensities, have recently been summarized elsewhere.^19,96^

To further analyze the nature of the IR bands, anharmonic calculations of the intensity were performed considering mechanical (wave functions) or electrical (electric dipole) anharmonicity, separately as well as together. In addition, the effect of resonances on the intensity redistribution was investigated by comparing the results obtained from GVPT2 with those of IDVPT2, where variational corrections were not included. This allowed us to better understand the impact of the GVPT2 model and the hybrid PES/PS scheme used on the intensity patterns. It should be emphasized that such computations are performed solely for the purpose of dissecting different effects and contributions. GVPT2 computations, including both mechanical and electrical anharmonicities, with the inclusion of all terms at the VPT2 or subsequent variational steps, represent the most complete framework and thus should be used for comparison with experimental results and spectral predictions.

In the manuscript, the numbering of the normal modes will follow the spectroscopic order, that is, by grouping vibrations based on their irreducible representation, and inside a given set, by decreasing order of energies. For the sake of readability, the harmonic and (I)­DVPT2 states will be described as a list of excited modes, with the number of quanta for a given mode indicated in superscript. In the body of the text, the ket delimiter representing a state will be dropped, so “4^1^6^1^” would represent a harmonic or (I)­DVPT2 binary combination of modes 4 and 6 with 1 quantum associated with each mode. GVPT2 states are indicated with a single index number, which corresponds to their position in the full list of eigenvalues, sorted by increasing order in energy (state 1 being the state with the lowest energy).

## Results and Discussion

### Equilibrium Structure and Harmonic Spectral Parameters

The equilibrium geometry of uracil is depicted in Figure 1, while the structural parameters optimized at the revDSD-PBEP86-D3­(BJ)/jun-cc-pVTZ and B3PW91-D3­(BJ)/jun-cc-pVTZ levels are reported in Table S1 in the Supporting Information (SI). The quality of DFT structures can be assessed by comparing with the reference ones determined theoretically from composite schemes of increasing accuracy,^31,66,97^ or from semiexperimental approaches employing experimental data from high-resolution microwave studies^98,99^ or gas-phase electron diffraction (GED).^66^ These experimental and theoretical results are in very good agreement with each other, with average deviations for the bond lengths of 0.002 Å and the largest deviation being 0.007 Å. DFT structures computed with both revDSD-PBEP86-D3­(BJ) and B3PW91-D3­(BJ) are close to those reference data (see Table S1), showing bond length deviations with MAEs lower or equal 0.0035 Å and maximum discrepancies (|MAX|) not exceeding 0.008 Å. Compared to the most recent and most accurate semi-experimental data estimated by Császár et al.,^97^ both methods show average errors below 0.003 Å, while revDSD-PBEP86-D3­(BJ) also shows |MAX| within 0.005 Å.

Slightly larger variations are observed between the literature data for the angles. For example, the average and largest deviation between the GED data and the semiexperimental data from Császár et al. reach 0.6° and 3.0°, respectively. The other reference data are more consistent with average differences not exceeding 0.15° and maximum differences within 0.26°. DFT results are again comparable to the best estimates. revDSD-PBEP86-D3­(BJ) performs better, showing MAE and |MAX| with respect to semiexperimental data by Császár et al.^97^ of 0.11 and 0.26°, respectively, while for B3PW91-D3­(BJ), the corresponding errors are 0.16° and 0.40°, respectively. Overall, both revDSD-PBEP86-D3­(BJ) and B3PW91-D3­(BJ) yield equilibrium structures accurate enough for the calculation of the harmonic and anharmonic force constants.

Harmonic computations at the revDSD-PBEP86-D3­(BJ) level have also been employed for the PED analysis and the description of the normal modes, as shown in Table S2 of the SI. PED results are consistent with the ones reported by Yarasi et al.^100^ Uracil is also one of the few cases for which harmonic wavenumbers and IR intensities have been obtained with the “cheap” composite scheme, where an extrapolation to the basis set limit, as well as core–valence correlation and diffuse-function corrections, have been considered by means of the second-order Mo̷ller–Plesset perturbation theory.^33^ The harmonic values computed at the revDSD-PBEP86-D3­(BJ) and B3PW91-D3­(BJ) levels are compared with the values from ref 33 in Table S3 of the SI. revDSD-PBEP86-D3­(BJ) is clearly in better agreement, with a MAE for the energies of 6 cm^–1^ and a maximum error of 17 cm^–1^ (for mode 5), compared to 9 and 30 cm^–1^ (for mode 27) for B3PW91-D3­(BJ). Importantly, both revDSD-PBEP86-D3­(BJ) and B3PW91-D3­(BJ) show relatively large errors for modes 5 and 6, where the leading contributions are the νC2O (68%) and νC4O (66%) stretching motions, respectively. For revDSD-PBEP86-D3­(BJ), the errors for these modes are 17 and 12 cm^–1^, while for B3PW91-D3­(BJ), they are 26 and 21 cm^–1^. Since these two transitions are the most intense and involved in Fermi resonances (vide infra), we anticipate that such deviations could result in noticeable differences in the spectral patterns.

Considering now the intensities, revDSD-PBEP86-D3­(BJ) agrees better with the reference, producing an MAE of about 7 km/mol and |MAX| of about 40 km/mol, still clearly outperforming B3PW91-D3­(BJ), which has MAE and |MAX| of 13 and 123 km/mol, respectively. Interestingly, both methods show the largest deviations for close transitions, that is, the fundamentals of modes 5 and 6 and those of modes 9 and 10. However, revDSD-PBEP86-D3­(BJ) qualitatively agrees with the reference, showing similar intensity for doublet 5–6, while predicting the fundamental of mode 9 to be more intense than mode 10. At variance, B3PW91-D3­(BJ) predicts the fundamental of mode 6 to be 20% more intense than that of mode 5 and reverses the intensity order between modes 9 and 10. It should also be noted that the nature of modes 9 and 10 is more complex to characterize (see Table S2), with the leading contributions from δ­(NH), δ­(CH), and ν­(CC), where δ and ν denote bending and stretching motions in the plane, respectively. Mode 9 is composed of 22% from δ­(N1H), 14% δ­(C6H), 12% ν­(C4C5) and 12% ν­(C2C3), and mode 10 is composed of 26% δ­(N3H), 15% δ­(C6H), 13% δ­(C5H) and 12% ν­(C1C2). Thus, the larger errors can also be due to the lower similarity of normal modes with respect to the reference.

This comparison with the reference data confirms the higher accuracy of the revDSD-PBEP86-D3­(BJ) computations, suggesting that the harmonic part should be performed at least at this level. For this reason and considering the need to balance the overall computational cost, the hybrid revDSD-PBEP86-D3­(BJ)/B3PW91-D3­(BJ) scheme was adopted for full anharmonic calculations.

### Reliable Description of Anharmonic Intensities

#### Changes in Spectral Line-Shape Due to Anharmonic Effects

To better understand the sensitivity of the spectral band shape to the methodology used to derive hybrid PES and PS, we consider the three types of hybrid schemes previously described, labeled “Freq”, “PES”, and “PES+PS”, for which sample input files are reported in Tables S4–S6 in the SI.

The anharmonic effects on the overall IR spectrum are analyzed considering intensity redistribution caused by the variational correction by comparing the IDVPT2 and GVPT2 spectra, as well as in terms of mechanical and electrical contributions to the anharmonic correction. The latter are analyzed at the IDVPT2 level to avoid any influence from the mode mixing. We should mention that fundamental bands are governed by 4 types of contributions: harmonic, pure electrical, pure mechanical, and mixed electrical-mechanical. By considering only one anharmonic contribution at a time, expanding either the potential energy or the property beyond the harmonic limit, we obtain two additional spectra. The “mechanical” spectrum thus shows the harmonic and pure mechanical contributions, neglecting any higher-order contributions from the property, while the “electrical” one includes the harmonic and pure electrical contributions. In both cases, the contributions from the mixed term are null. It should be mentioned that, typically, the harmonic term is largely dominant. For the first overtones and ‘1+1’ binary combinations (i.e., combinations of two modes), there are no harmonic contributions, and no mixed term, so the only contributions are purely mechanical or electrical. In this particular case, combining the band shapes of each individual contribution should result in the “full” band shape. More details on the actual formulas can be found in ref 53.

To facilitate the study of the shape of the IR spectral band, the latter is split into five regions, starting from the lowest energies: (i) below 1000 cm^–1^; (ii) from 1000 to 1800 cm^–1^, the typical fingerprint region; (iii) from 1800 to 2900 cm^–1^, a region dominated by nonfundamental bands; (iv) from 2900 to 3600 cm^–1^, the CH and NH stretching region; (v) above 3600 cm^–1^, the near-infrared (NIR) region.

The low-energy region below 1000 cm^–1^, reported in Figure S1 in the SI, is dominated by fundamental transitions. The line shapes in this region obtained with all hybrid schemes are rather similar. The most visible difference is for the band at about 550 cm^–1^, which shows a rich structure comprising three fundamentals, 18^1^, 27^1^, and 19^1^ for “PES+PS” and “PES”, while only two bands can be distinguished for “Freq” related to the fundamental transitions of modes 27 and 19. The difference is caused by the position of 18^1^, which is closer to 27^1^ in the latter case, resulting in a single peak. This effect is enhanced for GVPT2, since, for all hybrid schemes, the position of 18^1^ is red-shifted with respect to its IDVPT2 counterpart. For the low-intensity bands at around 685–700 cm^–1^, two transitions assigned to the combinations 30^1^27^1^ and 30^1^18^1^ lie closer to each other for the calculations of “Freq” due to a blue shift of the former and a red shift of the latter, leading to one broad band. Conversely, “PES” and “PES+PS” show two distinct peaks. With all hybrid models, states 30^1^18^1^ and 30^1^19^1^ gain intensity due to their interaction with the fundamental of mode 26, while the position of all three bands within 685–700 cm^–1^ remains unchanged from that of IDVPT2.

Looking at the contributions to the total anharmonicity of the most visible nonfundamental bands at the IDVPT2 level, we observe that the intense combinations 28^1^27^1^ and 30^1^27^1^ are characterized by a constructive role of their electrical and mechanical components, while for some lower intensity ones such as 30^1^29^1^, the larger mechanical contribution is partially canceled by the electrical component.

Switching to the fingerprint region (1000–1800 cm^–1^), the band shape is reported in Figure 2. Further details on the lower-intensity and denser regions are provided in Figure S2 in the SI. At the IDVPT2 level, the intense bands correspond to fundamental transitions, among which 5^1^ and 6^1^ (C=O stretching vibrations) are dominant regardless of the hybrid scheme. Nevertheless, some differences can be observed between these two bands depending on the model. They are of comparable intensity for “PES+PS”, while both “Freq” and “PES” show 6^1^ being about 10% more intense. The spectral pattern in the 1690–1800 cm^–1^ range changes qualitatively at the GVPT2 level due to Fermi resonances, as will be discussed in more detail below. Notable contrast between the intensity pattern generated with different hybrid schemes for the 1380–1400 cm^–1^ range will also be analyzed in the next section.

**Figure 2:**
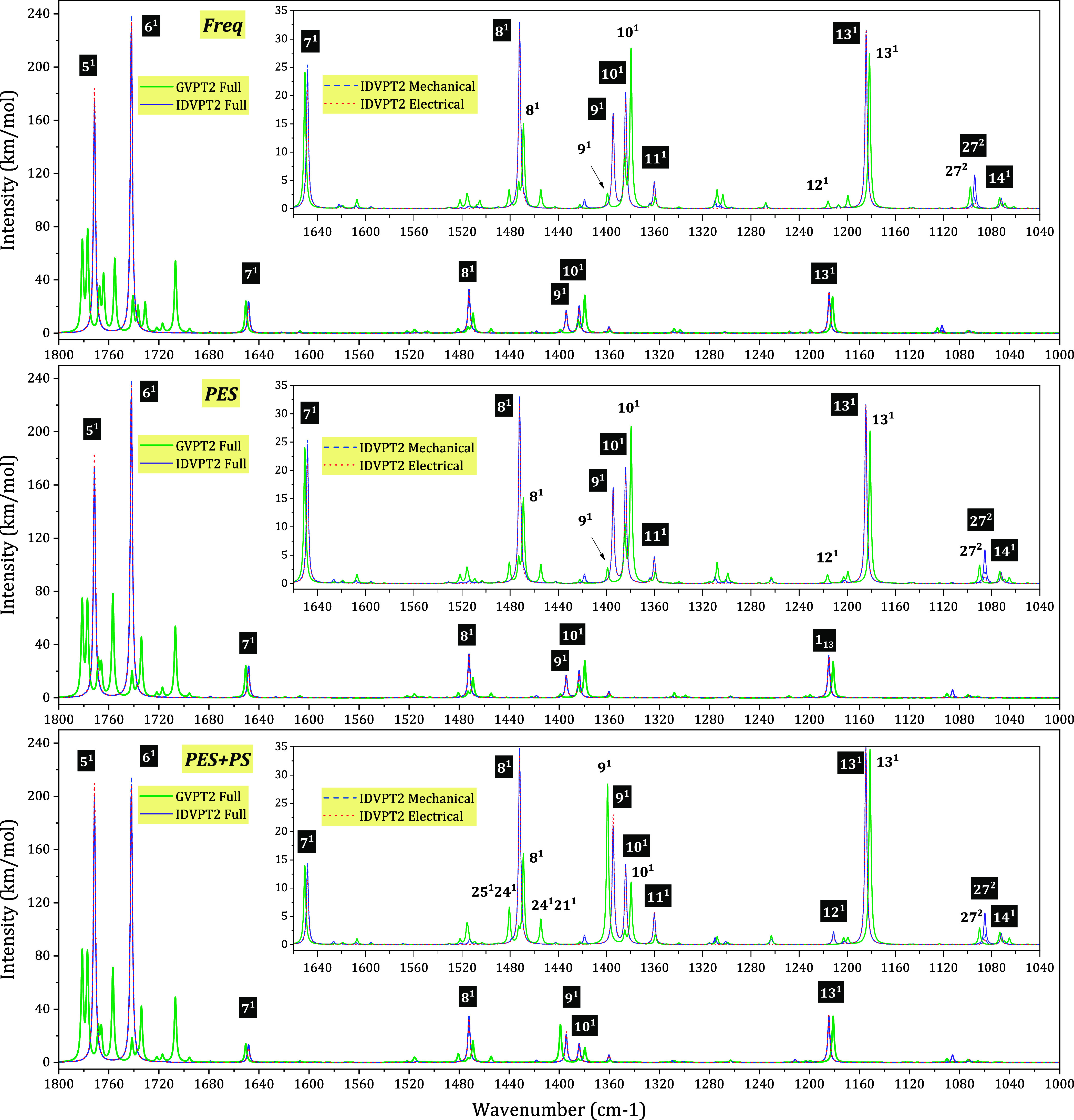
Theoretical IR spectra with different revDSD-PBEP86-D3­(BJ)/B3PW91-D3­(BJ) hybrid schemes for the 1000–1800 cm^–1^ region. The simulated spectra are modeled by applying Lorentzian distribution functions with half-widths at half-maximum = 1 cm^–1^ and a grid step of 0.1 cm^–1^ to each computed transition. Most relevant bands are assigned, with the assignment for IDVPT2 shown as white on black.

A closer look at the less intense bands in the fingerprint region is shown in the insets in Figures 2 and in S2 in the SI. For all hybrid models, the fundamental 8^1^ loses intensity at the GVPT2 level due to many resonances within the polyad, as indicated in Figure 3. However, its distribution among several nonfundamental bands via variational correction varies for each hybrid computation, from “Freq”, showing a more uniform intensity pattern, to “PES+PS”, where three bands 24^2^, 25^1^24^1^, and 24^1^21^1^ are the most outstanding. 26^1^23^1^, which appears as a shoulder of the band associated with 8^1^, is more intense for “Freq” and “PES” than for “PES+PS”. The differences between hybrid models are also due to 9^1^ and 10^1^ (vide infra). Overall, most nonfundamental bands in this range gain intensity at the GVPT2 level, except 27^2^, which is relatively intense already at the IDVPT2 level, thanks to large constructive mechanical and electrical contributions. Among the fundamental bands, 7^1^ is the least affected by the variational correction, but its intensity still varies slightly, being the lowest for “PES+PS”. For nonfundamental transitions, only a few are not affected by resonances, for instance, 27^1^25^1^, with a position relatively far from any fundamental transition. This combination band is most intense for “PES+PS” due to the increased mechanical component. Overall, it is evident that in the 1000–1800 cm^–1^ range, intensity redistribution through resonances plays a critical role, and so does mode coupling. The intensity distributions are also strongly affected by the choice of hybridization scheme, with “Freq” and “PES” yielding mostly similar results and “PES+PS” exhibiting the largest differences.

**Figure 3:**
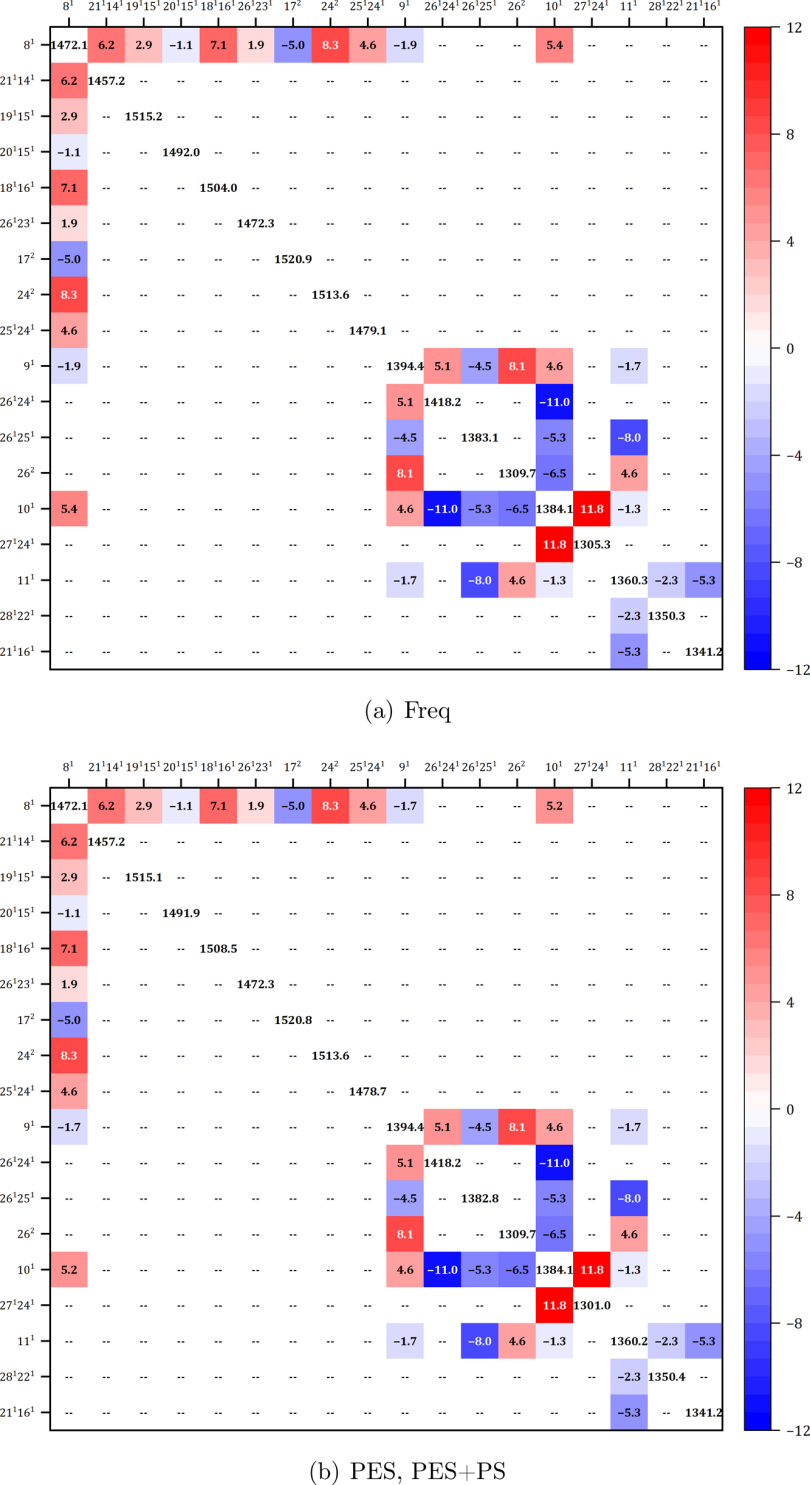
Variational matrix involving fundamentals 9^1^ and 10^1^, as computed at the revDSD-PBEP86-D3­(BJ)/B3PW91-D3­(BJ) level. (a): hybrid scheme “Freq”, (b): hybrid schemes “PES”, “PES+PS”.

The 1800–2900 cm^–1^ region is shown in Figure S3 in the SI and consists of overtones and combination bands that are totally free from resonances; thus, frequencies and intensities are identical for IDVPT2 and GVPT2. The patterns predicted by all hybrid schemes are quite similar in this range, except for the bands around 2150 cm^–1^, where the inclusion of dipole moment derivatives in “PES+PS” lowers the intensities. The intensities for most bands in the 1800–2250 cm^–1^ range are dominated by the mechanical component. In the range 2250–2900 cm^–1^, the contributions are more balanced, while the electrical component dominates only in a few cases, among which 22^2^ at about 1920 cm^–1^ is the most outstanding.

In the region of fundamental C–H and N–H stretching vibrations (2900–3600 cm^–1^, Figure S4 in the SI), the two strongest bands, 1^1^ and 2^1^ at 3490 and 3443 cm^–1^, respectively, are not affected by resonances. The lower-energy range at about 3000–3150 cm^–1^ includes 3^1^ and 4^1^ and nonfundamental states in resonance with them. Other overtones and combinations are less affected by the resonances. The differences between the hybrid schemes in this region are negligible.

Finally, the higher-energy nonfundamental transitions are included in the region above 3600 cm^–1^, extending further into the near-infrared (Figure S5 in the SI). Higher-quanta transitions are expected to gain more importance when reaching the NIR region. To check if this was the case here, 3-quanta transitions were also included in VPT2 calculations (cf. Figure S6 in the SI). For the range of interest here, their contributions are weak except for the part near 3600 cm^–1^, in particular an intense band at 3602 cm^–1^ composed of 17^1^14^1^5^1^ and 16^1^13^1^8^1^ transitions. To keep the discussions focused on the important features of the spectral band shapes, fundamentals, and 2-quanta transitions will be considered in the following. In the 5300–6000 cm^–1^ range, no relevant transitions are observed, so this region is not reported in Figure S5. As expected, no fundamental transitions are present above 3600 cm^–1^, which excludes any possibility of intensity redistribution from them. Hence, for all bands in this region, the hybrid schemes yield essentially the same spectral pattern, showing also the same variations of the relative intensity contributions. In the 3600–5300 cm^–1^ range, most bands are dominated by the electrical component. The most notable exceptions are 26^1^2^1^ and 27^1^1^1^, whose intensities are primarily governed by mechanical anharmonicity. Regarding the intensity of the stretching-mode overtones, those related to N–H (1^2^ and 2^2^) are mainly influenced by the mechanical anharmonicity, while for C–H, the electrical contributions dominate. The range of 4650–5000 cm^–1^ encompasses the most visible differences between the hybrid schemes. The total intensities of “PES+PS” are larger for combination bands 13^1^1^1^, 11^1^2^1^, 11^1^1^1^, 9^1^1^1^, and 8^1^1^1^ at about 4669, 4795, 4847, 4877, and 4959 cm^–1^, respectively. The two strongest ones (8^1^1^1^, 4959 cm^–1^ cm^–1^ and 11^1^2^1^, 4795 cm^–1^) are almost equally intense for “PES+PS”, while for the other two hybrid schemes, the intensity of the higher-energy band is approximately 70% that of the lower-energy one. We also notice an increase in the total intensity of the 26^1^2^1^ band at about 4094 cm^–1^ for “PES+PS”, compared with the other two hybrid schemes. This leads to different relative intensities of 26^1^2^1^ with respect to 27^1^1^1^ at about 4011 cm^–1^. However, these differences in the spectral patterns are much smaller than in regions where interactions between fundamental and nonfundamental transitions are possible.

To conclude this first part of the discussion, IDVPT2 computations allow for the analysis of mechanical and electrical contributions to the overall anharmonicity for all bands, while comparison between IDVPT2 and GVPT2 highlights which fundamental and nonfundamental transitions are involved in the anharmonic resonances and intensity redistributions. Coupled with the choice of the hybrid scheme, these results strongly affect the spectral pattern, especially in the fingerprint region. In order to provide further insights into the effects arising from the anharmonic resonances and those due to the hybrid schemes, we will focus on two specific transitions in that region and analyze the contributions to their energies and intensities.

#### Effect of the Hybrid Scheme on Relative Intensities: Bands 9^1^ and 10^1^

To analyze the impact of hybrid force fields and anharmonic contributions on the band positions and relative intensities, two close transitions at about 1400 cm^–1^, associated with fundamentals 9^1^ and 10^1^, were chosen. These transitions have been selected as they represent a clear doublet and are not expected to be involved in many anharmonic resonances. The experimental spectrum shows a well-defined pattern with the band at 1400 cm^–1^ stronger than the one at 1390 cm^–1^.^63^

Based on the potential energy distribution, both 9^1^ and 10^1^ can be described as combinations of C–H, N–H and C=O in-plane bendings, and bond stretchings of the ring, of which δ­(N1H) and δ­(N3H) are the leading contributions for 9^1^ and 10^1^, respectively (see Table S2 in the SI). Their harmonic IR intensities were computed at the revDSD-PBEP86-D3­(BJ) level (Table S3), of 72.0 and 42.8 km/mol, respectively, which match well the experimental pattern, while the B3PW91-D3­(BJ) computations yield more similar intensities for both modes with reversed order. Among GVPT2 revDSD-PBEP86-D3­(BJ)/B3PW91-D3­(BJ) computations, “Freq” and “PES” hybrid schemes show a similar and wrong intensity pattern compared to the experimental spectrum,^63^ while only “PES+PS” gets a correct intensity distribution, as illustrated in Table 1 and Figure 4.

**Table 1:** Wavenumbers (cm^–1^) and IR Intensities (km/mol) of Bands 9^1^, 10^1^ Computed at Different Levels^a^

	Harm					IDVPT2				GVPT2			
	9^1^		10^1^			9^1^		10^1^		9^1^		10^1^	
	ω	IR	ω	IR		ν	IR	ν	IR	ν	IR	ν	IR
PES+PS	1428.8	72.0	1418.1	42.7	full	1394.4	65.5	1384.1	43.4	1399.1	89.2	1379.5	34.3
					ele	1394.4	72.0	1384.1	41.9	1399.1	92.4	1379.5	26.3
					mcn	1394.4	65.7	1384.1	42.3	1399.1	88.3	1379.5	33.1
PES	1428.8	54.4	1418.1	61.6	full	1394.4	52.5	1384.1	63.3	1399.1	8.3	1379.5	86.1
					ele	1394.4	51.6	1384.1	60.4	1399.1	8.7	1379.5	76.1
					mcn	1394.4	52.4	1384.1	61.9	1399.1	8.6	1379.5	84.3
Freq	1428.8	54.4	1418.1	61.6	full	1394.4	52.4	1384.1	63.3	1399.1	8.2	1379.6	88.2
					ele	1394.4	51.6	1384.1	60.4	1399.1	8.7	1379.6	78.1
					mcn	1394.4	52.3	1384.1	61.8	1399.1	8.5	1379.6	86.3

a full, ele, mcn: Full, electrical, and mechanical anharmonicities.

**Figure 4:**
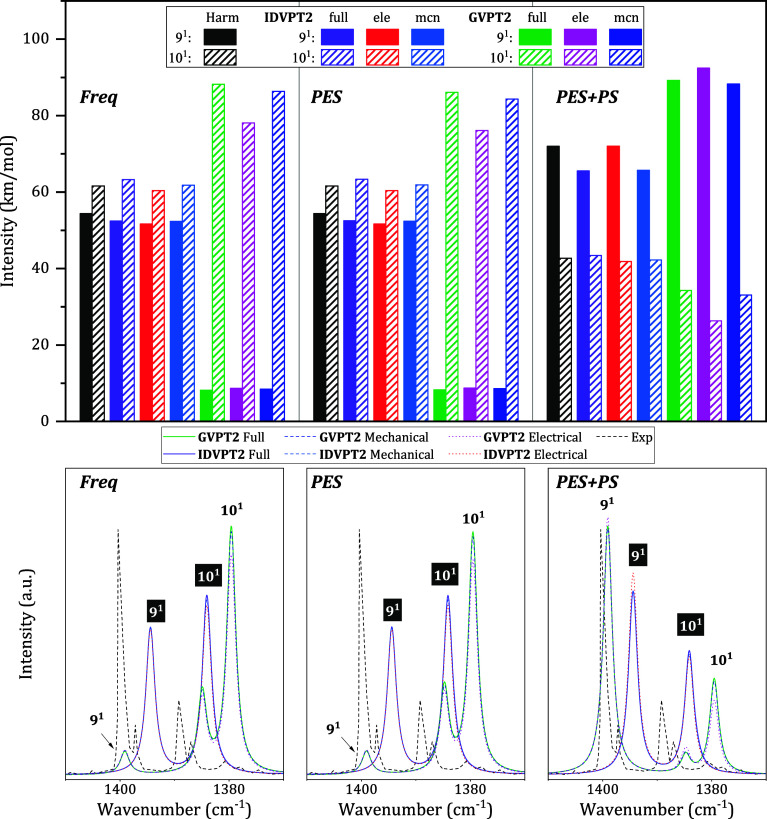
Comparison of intensities of 9^1^ and 10^1^ when combining revDSD-PBEP86-D3­(BJ) and B3PW91-D3­(BJ) using different schemes (labeled as “Freq”, “PES” and “PES+PS”), also considering different anharmonicities (full: Full, ele: electrical, mcn: mechanical), see top panel. The lower panels show a detail of the simulated IR spectra in the 1370–1410 cm^–1^ region, including 9^1^ and 10^1^. Experimental spectrum was registered in argon matrix by Ivanov et al.^63^ The simulated spectra are modeled by applying standard Lorentzian functions with a half-width at half-maximum = 1 cm^–1^ and grid step of 0.1 cm^–1^. Most relevant bands are assigned, with those related to IDVPT2 shown as white on black.

The discrepancy is solely related to the intensities; all schemes yield similar energies, of 1399 and 1380 cm^–1^, in good agreement, within 10 cm^–1^ or better, with the experiment. Table 1 reports the results obtained when mechanical or electrical anharmonicities are considered separately, leading in all cases to the same pattern as the full anharmonic computations. For the “Freq” and “PES” hybrid models, intensities very similar to harmonic B3PW91-D3­(BJ) computations are obtained at the IDVPT2 level, while the variational correction further enhances an inverted intensity pattern, leading to intensities that are very low for 9^1^ and strong for 10^1^. The fact that the harmonic part dominates at the IDVPT2 level is also indicated by the very small differences observed among “full”, “electrical,” and “mechanical” intensities. For “PES+PS”, the more correct distribution of harmonic revDSD-PBEP86-D3­(BJ) IR intensities is maintained with IDVPT2 and further improved after variational correction. The significant changes observed for all models when switching to GVPT2 can be explained by the fact that although modes 9^1^ and 10^1^ are not involved in strong resonances, they are still coupled with each other, as well as with a group of nonfundamental states involving modes 24 to 27 (see Figure 3). Moreover, both are coupled with the fundamental 8^1^, which, in turn, is connected to additional overtones and combination bands. This leads to some intensity redistribution within the resulting polyad, and the coherence between energy and property derivatives becomes crucial.

#### Effect of the Hybrid Scheme on Anharmonic Resonances

The most characteristic and complex features of uracil IR spectrum over the range considered in the present study lie in the 1690–1800 cm^–1^ region, which shows a multitude of strong IR bands,^62,77–79^ whose intensities can be traced back to multiple resonances involving two fundamental C=O stretching vibrations, 5^1^ (68% ν­(C2O)) and 6^1^ (66% ν­(C4O)). An analysis of the variational matrix (Figure 5) indicates that these two vibrations are not coupled directly, but each of them is coupled to several nonfundamental ones. Namely, both 5^1^ and 6^1^ are resonant with combination bands 18^1^12^1^, 17^1^15^1^, 18^1^13^1^, 20^1^12^1^, 17^1^16^1^; 5^1^ is additionally coupled to 21^1^9^1^, 21^1^10^1^, and 20^1^13^1^, while 6^1^ is resonant with 19^1^12^1^, 19^1^13^1^, and 21^1^11^1^. These coupling patterns and specific off-diagonal matrix elements are essentially the same for all of the hybrid force fields. The only differences are in the diagonal part, where the “Freq” IDVPT2 energies for states 18^1^12^1^ and 18^1^13^1^ are about 5 cm^–1^ lower than with the other two schemes.

**Figure 5:**
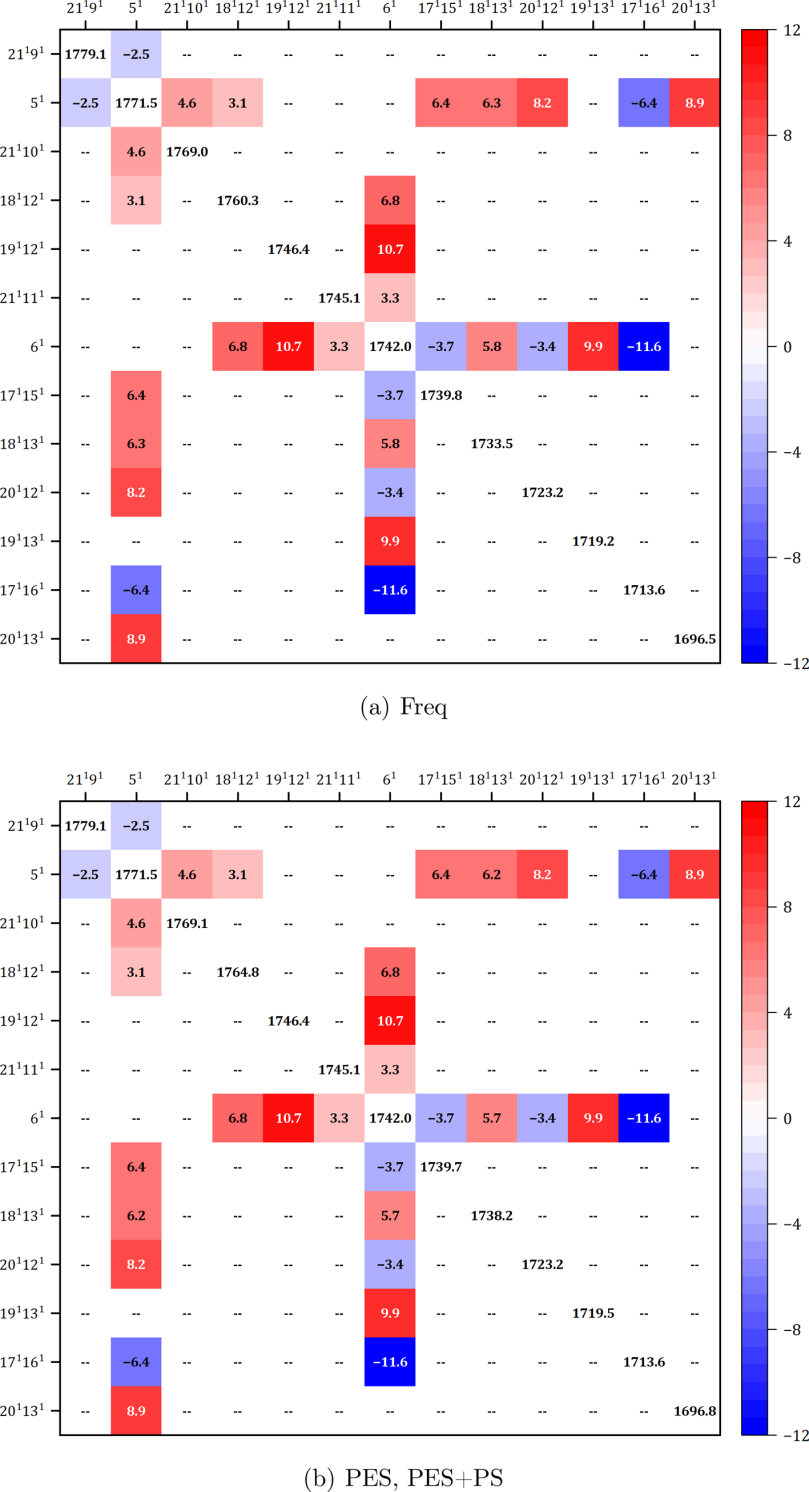
Variational matrix including fundamentals 5^1^ and 6^1^, as computed at the revDSD-PBEP86-D3­(BJ)/B3PW91-D3­(BJ)­level. (a): hybrid scheme “Freq”, (b): hybrid schemes “PES”, “PES+PS”.

The relevant part of the “PES+PS” normalized simulated spectrum is compared to experimental ones in Figure 6, while its “PES” and “Freq” counterparts are shown in Figure S7 in the SI. More details on the band assignment are provided in Tables 2 and S7 in the SI. The anharmonic vibrational energies of all bands in this range are the same for “PES” and “PES+PS”. The differences observed for “Freq” originate primarily at the IDVPT2 level (see Figure 5), but the impact on energies is less than 2 cm^–1^. For all hybrid schemes, IDVPT2 consistently shows only three bands, all due to fundamental transitions, while GVPT2 presents a richer pattern, caused by the intensity redistribution from the variational correction, as evidenced in Figure S2.

**Figure 6:**
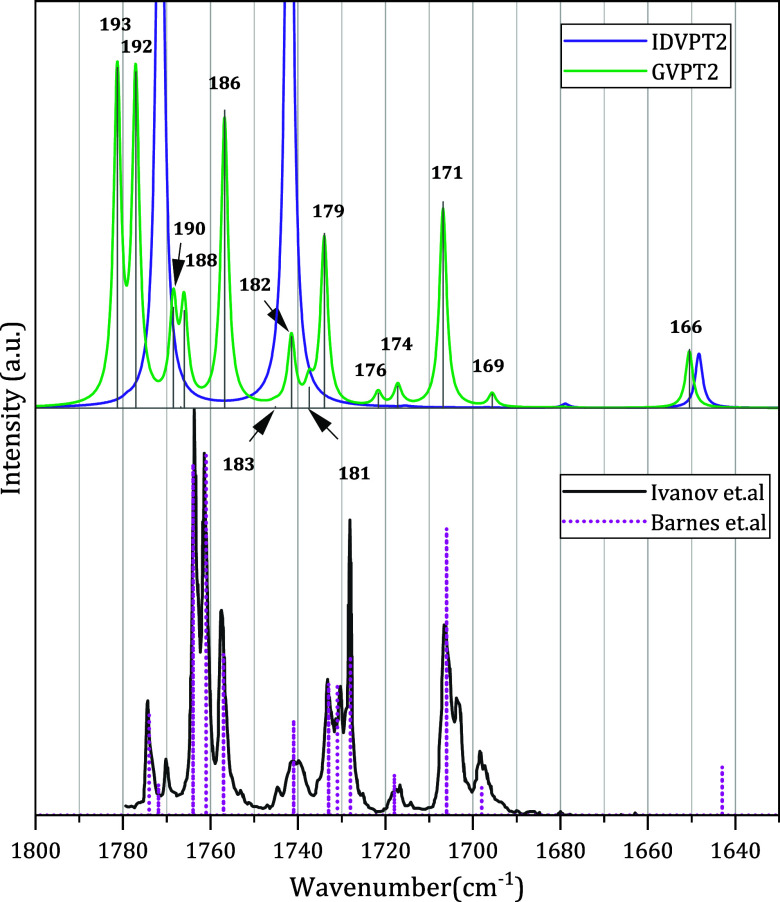
Anharmonic IR spectrum at the GVPT2 and IDVPT2 levels, in the 1600–1800 cm^–1^ region, along with experimental spectra from Ivanov et al.^63^ and Barnes et al.^62^ The simulated spectra are modeled with Lorentzian functions with a half-width at half-maximum of 1 cm^–1^ and a grid step of 0.1 cm^–1^.

**Table 2:** Calculated Anharmonic Wavenumbers and IR Intensities of Bands in the 1600–1800 Cm^–1^ Region Compared with Experimental Data^a^ ^,^ ^b^ ^,^ ^c^

	CVPT2^d^		current work - GVPT2 "PES + PS"					
exp.	ν	IR	state	ν	full	ele	mcn	polyad description
1764(vs)^e^	1761	364	193	1781	253.4	287.1	262.3	0.701 |21^1^9^1^⟩ −0.63 |5^1^⟩ – 0.235 |21^1^10^1^⟩
1761(vs)^e^	1753	77.6	192	1777	250.1	244.5	258.4	0.708 |21^1^9^1^⟩ + 0.567 |5^1^⟩ + 0.323 |21^1^10^1^⟩
1774(s)^e^	1776	20.2						
	1767	112	190	1769	74.9	83.1	80.7	0.673 |21^1^10^1^⟩ −0.638 |18^1^12^1^⟩ – 0.304 |6^1^⟩
1762.8^f^	1758	10.5	**189**	1767	0.9	1.8	0.0	|23^1^22^1^⟩
1770.2^f^	1767	112	188	1766	72.5	79.1	76.2	0.622 |21^1^10^1^⟩ + 0.534 |18^1^12^1^⟩ – 0.42 |5^1^⟩ + 0.285 |6^1^⟩
1757(vs)^e^	1752	52.5	186	1757	221.7	224.4	223.1	0.578 |19^1^12^1^⟩ + 0.558 |6^1^⟩ – 0.485 |18^1^12^1^⟩
1741(s)^e^	1742	13.8	**183**	1745	1.0	1.1	1.0	0.943 |21^1^11^1^⟩ – 0.327 |19^1^12^1^⟩
1733(s)^e^	1736	65.1	182	1742	53.1	46.6	55.8	0.571 |19^1^12^1^⟩ + 0.526 |17^1^15^1^⟩ – 0.481 |18^1^13^1^⟩ −0.266 |6^1^⟩ + 0.244 |21^1^11^1^⟩
1731(s)^e^			**181**	1737	15.6	17.8	16.7	0.788 |17^1^15^1^⟩ + 0.501 |18^1^13^1^⟩
1728(vs)^e^	1730	131	179	1734	129.9	140.5	132.8	0.667 |18^1^13^1^⟩ – 0.429 |6^1^⟩ + 0.368 |19^1^12^1^⟩ – 0.292 |19^1^13^1^⟩ + 0.261 |17^1^16^1^⟩
1718(m)^e^	1721	24.0	**176**	1722	11.0	12.5	10	0.941 |20^1^12^1^⟩ + 0.273 |19^1^13^1^⟩
	1718	18.6	174	1717	16.4	16.5	16.7	0.821 |19^1^13^1^⟩ + 0.470 |17^1^16^1^⟩ – 0.222 |20^1^12^1^⟩
1706(vs)^e^	1711	29.4						
	1697	119	**171**	1707	153.5	147.6	157.8	0.809 |17^1^16^1^⟩ + 0.436 |6^1^⟩ – 0.340 |19^1^13^1^⟩
1698(ms)^e^	1688	15.1	**169**	1696	10.4	12.3	10.7	0.989 |20^1^13^1^⟩
1641(s)^g^	1643	27.6	**166**	1651	43.7	33.6	45.9	0.953 |7^1^⟩

a **Exp.**: Most reliable experimental data; Relative intensities are given in parentheses.

b **full, ele, mcn**: IR intensities (in units of km/mol) considering “full”, “electrical”, and “mechanical” anharmonicity, respectively.

c **State numbers in bold**: assignments of the calculated bands are consistent with Krasnoshchekov et al.^64^

d Theoretical results by Krasnoshchekov et al.^64^

e Argon matrix by Barnes et al.^62^

f Argon matrix by Ivanov et al.^63^

g Gas phase by Colarusso et al.^67^

A striking improvement at GVPT2 level is the correct prediction of the intense doublet experimentally measured around 1760 cm^–1^. All hybrid schemes are in qualitative agreement, blue-shifted by about 16–17 cm^–1^, with “PES+PS” yielding the highest intensities for GVPT2 (see Figure 2). Looking more closely at the nature of these bands, the associated variational states are made up of about 50% 21^1^9^1^ and 30–40% of 5^1^. Consequently, assigning each state to a harmonic representation becomes tentative. Because of the complex nature of the states, we chose to keep the variational notation, using the absolute energy index of the state for the assignment.

The intensities of two medium peaks between 1760 and 1770 cm^–1^ (states 188 and 190) are the largest for “Freq”, and their relative ratios vary depending on the hybrid schemes. For “PES+PS,” both are essentially equal, while for “Freq” and “PES,” one is more intense, the lower- or higher-energy one, respectively. These intensity changes can be traced back to the relative contributions of single transitions. For “Freq”, the higher-energy one shows a leading component, at 76% (21^1^10^1^), while the lower one is described as 44% of 18^1^12^1^ and 25% of 6^1^ (both also having minor contributions from 5^1^). For both “PES” and “PES+PS”, the mixing is stronger (Tables 2 and S7 in the SI), so both are characterized by significant contributions from combination transitions 21^1^10^1^ and 18^1^12^1^. However, these differences can be considered minor, and trying to assess the quality of the hybrid schemes by comparison with the experiment is more challenging for these bands. The next strong peak, state 186 for “PES” and “PES+PS” and state 185 for “Freq”, with predicted intensity similar to 5^1^ in all cases, can be related to the band at 1757 cm^–1^ in the experiment,^62^ which is rather strong but weaker than the main doublet. A likely reason for the excessively large predicted intensity is an incorrect prediction of the magnitude of the resonance between states 6^1^ and 19^1^12^1^, which could be caused by an overestimation of the harmonic wavenumber of mode 6 with respect to the best theoretical estimate.^33^ For the three peaks around 1725–1740 cm^–1^, the overall pattern with three bands of comparable intensity is quite different for “Freq” compared to the other two hybrid schemes. By comparison with experiment, it is clear that the lowest-energy band should be the most intense and accompanied by shoulder transitions, while the highest-energy one would be a separate, lower-intensity peak. “PES” and “PES+PS” predict a pattern consistent with these observations, with the lowest energy band having a mixed nature at the GVPT2 level, combining in a similar ratio 19^1^12^1^ and 6^1^, and to a lesser extent 18^1^12^1^. It should be noted that fundamental 6^1^, in all cases, contributes to several transitions but dominates in neither. The assignment of the multiplet on 1728–1733 cm^–1^ was determined in accordance with the literature.^64^ As a final remark, this pattern shifts to higher energies when changing the environment from an Ar to a Ne matrix.^63^ For the remaining bands involved in anharmonic resonances, all hybrid schemes show the same pattern, consistent with experiment.

Overall, the “Freq” and “PES­(+PS)” hybrid schemes result in only minor deviations in the vibrational energies at the IDVPT2 level. However, these variations are sufficient to significantly affect the description of the variational states. This leads to even greater changes in the GVPT2 intensities, while the GVPT2 energies may be barely affected. These results indicate that consistencies in the PES and PS using the same (higher or lower) ESQM level within a hybrid scheme are particularly important for a correct intensity pattern in the regions involving large variational corrections.

### Comparison with Experiment

A comprehensive analysis of the vibrational signatures of uracil is performed by comparing experimental data^62,63,67,69,75,77,78,101–103^ with the simulation results from the “PES+PS” hybrid model. Since the focus is on the IR intensities, the quality and completeness of the reported experimental spectra are crucial for the comparison. IR spectra measured in inert-gas low-temperature matrices have the advantage of narrow bands, suitable for dissecting single transitions and comparison with computations, despite the possibility of matrix environment effects. Among all results, the most detailed experimental IR spectrum shape measured in the argon low-temperature matrix by Ivanov et al.^63^ is used as a reference in Figure 6. However, such a detailed spectral shape is not reported for the whole MIR range, nor any quantitative information on the band intensities is provided in ref 63. Missing information can be obtained from other matrix-isolation experiments^62,77,78^ that report the data on the relative intensities of the main transitions. Among them, the data from Barnes et al.^62^ are used to support and complement the initial set. They are either reported as a stick spectrum in Figure 6 or reconvoluted to simulate the spectral shape in the regions when no such information would be available otherwise (Figures 7 and S7). A list of all relevant experimental data is provided in Table S8. It should be noted that there is no experimental reference for the regions covering only nonfundamental transitions either in the MIR or the NIR range, so the comparison with experiment will be only performed in three energy ranges, starting from highest energies: 2800–4000 cm^–1^, 1600–1800 cm^–1^ and 100–1600 cm^–1^.

**Figure 7:**
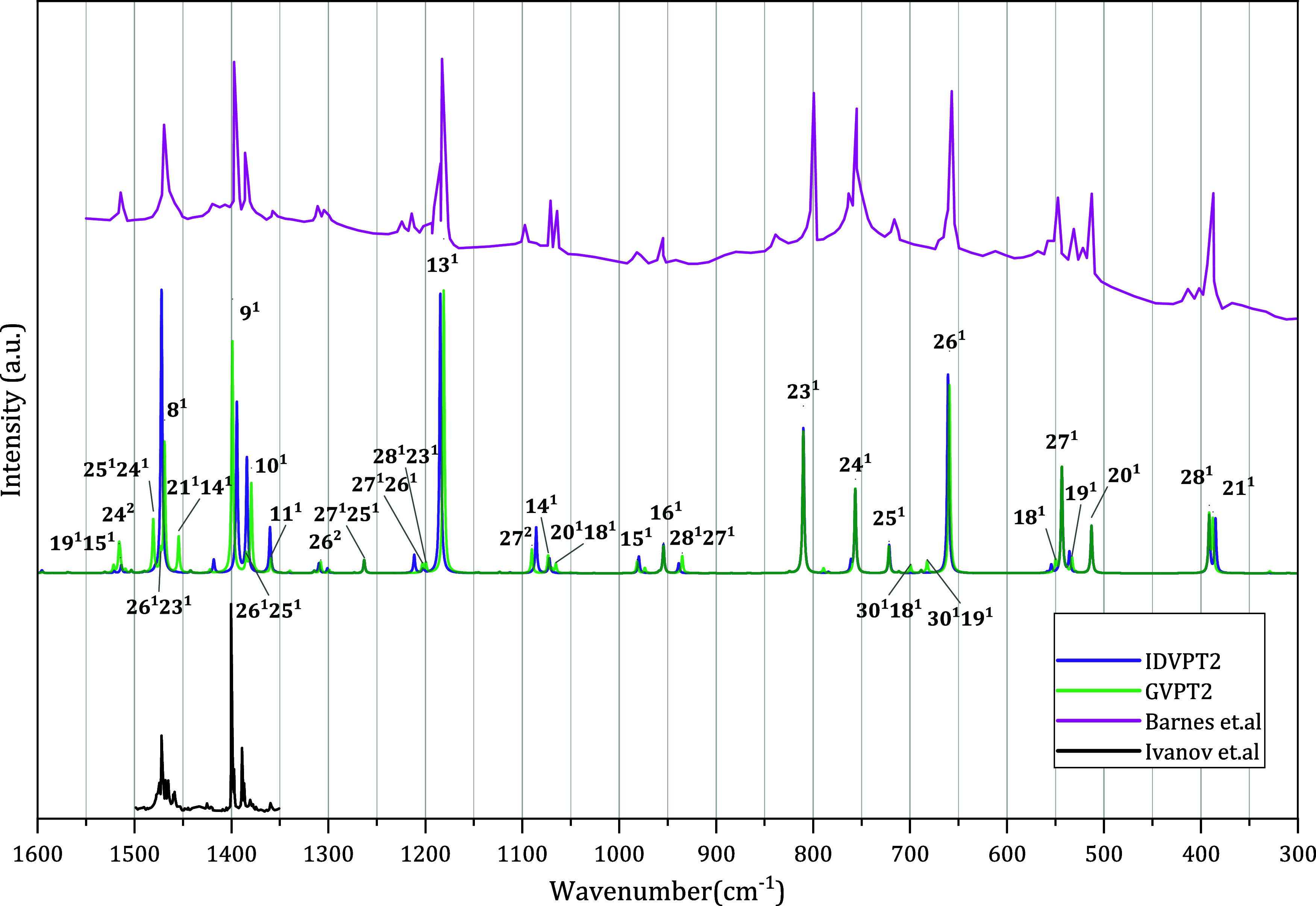
IR spectra in the 300–1600 cm^–1^ region. Anharmonic GVPT2 and IDVPT2 spectra computed at the revDSD-PBEP86-D3­(BJ)/B3PW91-D3­(BJ) level with the jun-cc-pVTZ, along with experimental spectra (Ivanov et al.^63^ and Barnes et al.^62^).

#### Spectral Range 2800–4000 cm^–1^

Experimental data, calculated anharmonic wavenumbers and IR intensities in the 2800–4000 cm^–1^ region, along with the definition of the states, are listed in Table 3. Interpretation of vibrational bands in this range is rather straightforward, with only two strong bands related to the 1^1^ (νN1H) and 2^1^ (νN3H) stretching vibrations. These bands have been measured in the gas phase,^67^ low temperature matrices^62,63,77,78^ and in helium nanodroplets,^102^ with all experiments agreeing within 10 cm^–1^. This work and CC/MP2 results by Krasnoshchekov et al.,^64^ predict 1^1^ and 2^1^ energies close to experimental values, within 6 cm^–1^. Integrated intensities reported in ref 102 allow also for direct comparison with computed values for the resonance-free fundamental transitions 1^1^ and 2^1^, showing agreement within 20%. Additional bands in this region are rather weak. Among them, there are two other fundamentals, 3^1^ (95% νC5H) and 4^1^ (95% νC6H), which are both involved in resonances with several combination bands, especially 8^1^7^1^ (see Table 3). Two other bands, observed at 3472.8 ^63^ and 2970 cm^–1^,^78^ respectively, are related to the 6^2^ overtone at 3468.9 cm^–1^ (3.1 km/mol) and the 11^1^7^1^ combination at 2998.5 cm^–1^ (0.7 km/mol). Overall, these weak transitions agree well with experiment, with discrepancies within 4–8 cm^–1^, and the only larger error of about 30 cm^–1^ is noted for the combination band observed at 2970 cm^–1^. These results are also consistent with previous anharmonic computations based on best-estimated harmonic frequencies employing either CC/DFT^33^ or CC/MP2 ^64^ hybrid schemes.

**Table 3:** Calculated Anharmonic Wavenumbers and IR Intensities of Bands in the 2800–4000 cm^–1^ Region Compared to Experimental Data^a^ ^,^ ^b^

	CVPT2^c^		current work - GVPT2 "PES + PS"					
exp.	ν	IR	state	ν	full	ele	mcn	polyad description
3484(s)^d^	3486	105	**385**	3490	93.7	105.5	99.9	|1^1^⟩
3472.8^e^	3435	63.5	382	3469	3.1	0.6	1.0	|6^2^⟩
3436(s)^d^			**381**	3444	60.2	65.7	63.4	0.992|2^1^⟩
			369	3148	1.2	1.6	1.8	0.724 |3^1^⟩ – 0.412 |8^1^7^1^⟩ + 0.400 |9^1^6^1^⟩ + 0.358 |4^1^⟩
			366	3123	1.2	0.2	0.5	|10^1^6^1^⟩
3124(m)^d^	3134	1.30	365	3119	0.6	0.2	0.2	0.653 |3^1^⟩ + 0.62.5 |8^1^7^1^⟩ – 0.31.3 |4^1^⟩
3084(m)^f^	3081	0.58	**363**	3093	1.7	1.0	0.7	0.709 |4^1^⟩ + 0.538 |8^1^7^1^⟩ – 35.3% |9^1^7^1^⟩
2970(15)^g^ ^,^ ^h^			360	2999	0.7	0.5	0.3	0.918 |11^1^7^1^⟩ – 0.300 |4^1^⟩

a **Exp:** Most reliable experimental data, relative intensities are given in parentheses.

b **State numbers in bold**: assignments of the calculated bands are consistent with Krasnoshchekov et al.^64^

c Theoretical results by Krasnoshchekov et al.^64^

d Spectrum in gas-phase measured by Colarusso et al.^67^

e Argon matrix experiment by Ivanov et al.^63^

f Argon matrix experiment by Barnes et al.^62^

g Argon matrix experiment by Szczesniak et al.^78^

h Relative intensity with respect to 2^1^ at 3433 cm^–1^, set at 100.^78^

#### Intense Anharmonic Transitions Due to Fermi Resonances in the 1600–1800 cm^–1^ Range

In this region rich in Fermi resonances, which can significantly alter the band shape, the quality of reference data is critical for a systematic comparison. An exhaustive list of experimental data has been built by combining multiple sources and is reported in Table 2 (see Table S8 in the SI for all experimental data). In the range of 1750–1800 cm^–1^, there are at least three very strong bands, 1764 (vs), 1761 (vs) and 1757 (vs),^62,63^ which are connected to the calculated ones at 1781.3 (253.4 km/mol), 1777.1 (250.1) and 1756.8 cm^–1^ (221.7). Among them, the first two are characterized by a strong mixture of 5^1^ and 21^1^9^1^ (see Table 2). They are blue-shifted with respect to the experiment by 17 and 16 cm^–1^, respectively. This discrepancy is traced back to the harmonic wavenumber of 5^1^, blue-shifted with respect to the best estimated theoretical value, as mentioned before. There are also two strong bands at 1774 cm^–1^ (s)^62,63^ and 1770.2 cm^–1^,^63^ which are assigned to states 190 and 188, both related to combinations 21^1^10^1^ and 18^1^12^1^, predicted at 1768.5 cm^–1^ (74.9 km/mol) and 1766.0 cm^–1^ (72.5 km/mol), respectively, so within 5 cm^–1^ of the experiment. Thus, our calculations successfully reproduce the five characteristic bands in the range of 1750–1800 cm^–1^ despite the significant blue shifts of the two very strong ones.

Comparing our results with those at the CVPT2//CC/MP2 level by Krasnoshchekov et al.,^57^ we note that the most intense transition, assigned to 5^1^ at 1761.2 cm^–1^ with an IR intensity of 364 km/mol, was predicted closer to the experimental band, in line with the harmonic wavenumber from the CC composite scheme.^33^ However, the use of the hybrid scheme of type “Freq” also led to a less reliable estimate of the intensity redistribution, with 5^1^ dominating, and 21^1^9^1^ 1 order of magnitude smaller (20.2 km/mol), instead of the doublet observed experimentally^62,63^ (see Figure 6).

Then, three strong bands are visible in the middle of the 1720–1750 cm^–1^ span, at 1733 (s), 1731 (s), and 1728 (vs) cm^–1^, respectively, as well as another weaker band at 1741 cm^–1^.^62,63^ The “triplet band” is well reproduced by transitions to 179, 181, and 182 states in simulations, with the most intense peak placed on its red side at 1734.0 cm^–1^ (129.9 km/mol). All states are the result of a complex polyad pattern. They are composed of 18^1^13^1^, with other significant contributions being 6^1^, 19^1^12^1^, and 17^1^15^1^. Polyad descriptions are different from those of Krasnoshchekov et al. to some extent. A likely explanation is the blue shift of about 12 cm^–1^ of the 6^1^ harmonic energy with respect to the CC best estimated one (see Table S3). This blue shift also affects the intensity of band 186 (combination of 19^1^12^1^ in strong resonance with 6^1^), leading to a more intense peak at 1757 cm^–1^ in this work. To complete this analysis, the band observed at 1741 cm^–1^ can be related to the computed state 183 at 1745.2 cm^–1^, but its intensity is predicted to be negligible (1.0 km/mol). This band is mainly composed of overtone 21^1^11^1^, in line with the assignment by Krasnoshchekov et al., which was also predicted at 1741.8 cm^–1^, but with a considerably higher intensity (13.8 km/mol), better matching the experiment.

The medium-intensity broader band reported at about 1718 cm^–1^ (m) is composed of several transitions, in line with calculated bands 176 at 1721.6 cm^–1^ (11.0 km/mol) and 174 at 1717.2 cm^–1^ (16.4 km/mol), both composed of 20^1^12^1^ and 19^1^13^1^. Another very strong band 171 observed at 1706 cm^–1^, is well reproduced at 1706.8 cm^–1^ (153.5 km/mol), and described by 17^1^16^1^ (65%) that strongly mixes with 6^1^ (19%). The band measured at 1698 cm^–1^ with medium intensity is linked to the combination band 169 (98% 20^1^13^1^) calculated at 1695.6 cm^–1^ (10.4 km/mol). These results and assignments are quite consistent with the ones by Krasnoshchekov et al., where 20^1^12^1^ (60%), 19^1^13^1^ (78%), 17^1^16^1^ (66%), and 20^1^13^1^ (89%) were computed at 1720.6, 1717.7, 1697.0, and 1688.2 cm^–1^, respectively. Finally, the third fundamental band at 1641 cm^–1^ (s) is well separated, essentially resonance-free, and corresponds to 7^1^, with the predicted position at 1650.5 cm^–1^ (43.7 km/mol) in this work and 1642.8 cm^–1^ (27.6 km/mol) by Krasnoshchekov et al.

Overall, we conclude that GVPT2 computations successfully reproduce the observed bands in the region 1600–1800 cm^–1^, despite the small blue shift of bands 192 and 193. These results highlight the sensitivity of the methods, and more generally, anharmonic calculations, to small differences in the description of underlying PES and PS in regions affected by strong resonances.

#### Spectral Range 100–1600 cm^–1^

This region involves fundamental bands 8^1^–30^1^, among which 8^1^–28^1^ have been observed by Fourier-transform infrared spectroscopy (FTIR) in the range of 1600–300 cm^–1^, and have been extensively documented in the literature.^62,75,78,101^ Figure 7 shows the simulated spectra compared to experimental plots^62,63^ in this region. The calculated energies and IR intensities of fundamental, overtone, and combination bands are listed in Table 4, along with the corresponding experimental data. Although the effect of Fermi resonances in this region is not as strong as above, several transitions are affected, gaining intensity from the redistribution. Nevertheless, the main peaks remain dominated by fundamentals (see Figure 7).

**Table 4:** Calculated Anharmonic Wavenumbers and IR Intensities of Bands in the Region below 1600 cm^–1^ Compared to Experimental Data^a^ ^,^ ^b^

	CVPT2^c^		current work - GVPT2 "PES+PS"					
exp.	ν	IR	state	ν	full	ele	mcn	polyad description
			142	1522	2.8	2.1	2.9	0.973 |17^2^⟩
1515(m)^d^			139	1516	9.7	3.2	10.2	0.687 |19^1^15^1^⟩ + 0.654 |24^2^⟩
			138	1515	5.1	1.0	5.2	0.719 |19^1^15^1^⟩ – 0.675 |24^2^⟩
			136	1509	1.3	1.2	1.6	0.958 |18^1^16^1^⟩ – 0.243 |24^2^⟩
			134	1503	1.0	0.8	0.2	|27^1^22^1^⟩
			128	1481	20.3	13.1	22.1	0.904 |25^1^24^1^⟩ + 0.377 |8^1^⟩
			127	1473	7.0	8.4	7.7	0.908 |26^1^23^1^⟩ + 0.304 |8^1^⟩ – 0.242 |25^1^24^1^⟩
1461(s)^d^	1463	81.1	**125**	1469	49.9	55.9	47.0	0.724 |8^1^⟩ −0.408 |26^1^23^1^⟩ + 0.382 |21^1^14^1^⟩ – 0.342 |25^1^24^1^⟩
1458.5^e^			122	1455	13.9	16.3	13.1	0.910 |21^1^14^1^⟩ – 0.395 |8^1^⟩
1422^f^			120	1422	1.3	0.4	0.9	0.952 |26^1^24^1^⟩ – 0.271 |10^1^⟩
1400(s)^d^	1394	37	**118**	1399	89.2	92.4	88.3	0.834 |9^1^⟩ + 0.396 |10^1^⟩ – 0.377 |26^1^25^1^⟩
1386(mw)^g^			117	1385	6.5	8.8	6.9	0.743 |26^1^25^1^⟩ + 0.495 |9^1^⟩ – 0.311 |10^1^⟩ −0.257 |11^1^⟩
1387(s)^d^	1382	69	**116**	1380	34.3	26.3	33.1	0.791 |10^1^⟩ + 0.433 |26^1^25^1^⟩ – 0.256 |11^1^⟩ + 0.248 |26^1^24^1^⟩
1356(m, sh)^d^	1351	7.66	**111**	1359	5.7	5.7	5.6	0.867 |11^1^⟩ + 0.335 |26^1^25^1^⟩ – 0.252 |21^1^16^1^⟩ – 0.221 |28^1^22^1^⟩
1313(vw)^g^			98	1308	4.3	0.2	2.9	0.981 |26^2^⟩
1304(vw)^g^			96	1299	1.3	1.7	0.4	0.983 |27^1^24^1^⟩
1283(25)^h^			90	1263	5.1	0.2	3.3	|27^1^25^1^⟩
			85	1216	0.1	0.6	0.3	0.900 |12^1^⟩ – 0.329 |28^1^27^1^⟩
1217(w)^g^	1214	2.00	83	1203	3.4	2.5	2.4	0.966 |27^1^26^1^⟩ + 0.222 |28^1^23^1^⟩
1192(vw)^i^			82	1199	3.7	3.0	2.6	0.911 |28^1^23^1^⟩ + 0.302 |12^1^⟩
1187(s)^d^	1180	98.8	**79**	1181	108.8	110.2	112.4	0.938 |13^1^⟩ + 0.308 |12^1^⟩
1082(m)^d^			63	1090	9.1	0.5	2.2	0.942 |27^2^⟩ – 0.285 |14^1^⟩
1073(w)^g^			60	1073	6.7	3.3	5.1	0.691 |19^2^⟩ + 0.617 |14^1^⟩ + 0.260 |20^1^18^1^⟩ + 0.228 |27^2^⟩
			59	1069	1.5	2.2	0.8	0.688 |19^2^⟩ – 0.519 |20^1^18^1^⟩ −0.473 |14^1^⟩
1066(w)^g^	1061	6.67	58	1065	3.5	2.0	3.0	0.813 |20^1^18^1^⟩ – 0.507 |14^1^⟩
984.7^e^	980	4.59	**52**	982	4.5	6.0	3.8	0.863 |15^1^⟩ + 0.430 |29^1^23^1^⟩
972(w, sh)^d^			51	974	2.0	1.4	2.3	0.884 |29^1^23^1^⟩ – 0.398 |15^1^⟩
958^g^	956	0.45	**50**	959	0.5	0.3	0.5	|22^1^⟩
952(w)^d^	948	10.4	**48**	954	9.9	8.7	11.0	0.965 |16^1^⟩
944(2)^f^			45	935	6.6	2.2	3.4	0.951 |28^1^27^1^⟩ – 0.247 |15^1^⟩
804(s)^g^ ^,^*	803	51.6	**32**	810	54.4	53.6	55.4	|23^1^⟩
792(2)^f^			30	789	1.8	1.0	1.2	0.921 |28^2^⟩ – 0.388 |17^1^⟩
761(s)^g^	752	1.29	27	757	30.6	30.0	31.4	|24^1^⟩
757(w)^d^	756	29.2	26	756	2.6	2.6	3.0	0.919 |17^1^⟩ + 0.384 |28^2^⟩
717(vw)^d^	716	8.92	**25**	722	10.4	11.0	10.6	0.990 |25^1^⟩
692(vw)^d^			21	700	2.9	2.5	2.5	0.984 |30^1^18^1^⟩
			20	689	1.1	0.4	0.2	|30^1^27^1^⟩
682(13)^h^			19	682	4.4	3.8	3.9	0.977 |30^1^19^1^⟩
662(s)^g^ ^,^^+^	651	32.4	**17**	659	72.2	71.0	72.7	0.972 |26^1^⟩
545(w)^d^	549	42.4	14	550	4.4	4.7	4.1	0.823 |18^1^⟩ + 0.527 |29^1^28^1^⟩
551(m)^g^			12	543	40.8	38.2	41.5	|27^1^⟩
538^j^	535	0.04	11	538	1.1	1.2	1.2	0.862 |30^1^28^1^⟩ + 0.481 |19^1^⟩
528^j^	531	7.44	**10**	533	5.6	4.4	5.2	0.838 |19^1^⟩ – 0.506 |30^1^28^1^⟩
512(w)^d^	511	16.1	**8**	513	18.3	19.8	19.0	|20^1^⟩
395(w)^d^	386	19.1	7	392	22.1	22.9	22.6	|28^1^⟩
374(vw)^d^	384	24.0	6	388	20.0	20.2	21.0	0.983 |21^1^⟩
166^j^ ^,^ ^k^	158	0.30	**2**	164	0.2	0.4	0.2	|29^1^⟩
149^l^	140	0.82	**1**	145	1.3	1.0	1.4	|30^1^⟩

a **State numbers in bold**: assignments of the calculated bands are consistent with Krasnoshchekov et al.^64^

b *,+: The most reliable experimental data are different with those chosen by Krasnoshchekov et al. (*: 802 (w)^d^, ^+^: 660 (w)^d^).

c Theoretical results by Krasnoshchekov et al.^64^

d Gas phase by Colarusso et al.^67^

e Argon or neon matrix by Ivanov et al.^63^

f Argon matrix by Leś et al.^101^

g Argon matrix by Barnes et al.^62^

h Argon matrix by Szczesniak et al.^78^

i Argon matrix by Malteseet al.^77^

j Aamouche et al.^69^

k Aamouche et al.^103^

l Fujii et al.^104^

The first group within 1420–1550 cm^–1^ is composed of two main bands accompanied by weak transitions. A stronger band measured at 1461 cm^–1^ ^67^ also includes a weak shoulder at 1458.5 cm^–1^. The main transition to state 125 (52% of 8^1^) is predicted at 1469.0 cm^–1^ with an intensity of 49.9 km/mol, and a shoulder at 1454.5 cm^–1^ (13.9 km/mol). These two transitions are in resonance and composed of 8^1^ and 21^1^14^1^, with the main band also having contributions from 26^1^23^1^ and 25^1^24^1^. A second, medium-intensity band at 1515 cm^–1^,^67^ is linked to two transitions computed at 1515.9 and 1514.8 cm^–1^, with intensities of 9.7 and 5.1 km/mol, respectively, assigned to 19^1^15^1^ and 24^2^, which are strongly resonant with each other, with nearly equal contributions (see Table 4). A weak band recorded at 1422 cm^–1^ ^101^ is predicted as 26^1^24^1^ (91%) at 1422.1 cm^–1^ (1.3 km/mol). Finally, some calculated bands with non-negligible intensities (larger than 1 km/mol), which have not been reported so far in the experiment, presumably due to a lack of theoretical data, are also listed in Table 4. For example, the combination band 25^1^24^1^ at 1480.7 cm^–1^ with an intensity of 20.3 km/mol should be visible experimentally. These results and assignments are consistent with the analysis by Krasnoshchekov et al., where, for instance, 8^1^ was predicted at 1462.7 cm^–1^ but, with an intensity of 81.1 km/mol, 60% larger than here.

In 1350–1400 cm^–1^ range, the bands observed at 1356 (m), 1387 (s), 1386 (mw) and 1400 cm^–1^ (s) are predicted at 1359.4 (5.7 km/mol), 1379.5 (34.3), 1384.8 (6.5) and 1399.1 cm^–1^ (89.2), and assigned to 11^1^ (75%), 10^1^ (63%), 26^1^25^1^ (55%) and 9^1^ (69%) respectively. The two most intense bands have been analyzed in detail in the validation part (see Figures 3 and S2b). For the above four bands, the deviations between theoretical and experimental data range from 1 to 8 cm^–1^, and the intensity distribution is well reproduced. The key fundamental bands 11^1^, 10^1^ and 9^1^ were predicted at 1351.0 cm^–1^ (7.7 km/mol), 1382.0 cm^–1^ (69.1 km/mol) and 1394.1 cm^–1^ (36.9 km/mol) by Krasnoshchekov et al., so showing good agreement for the energies, but with a reverse order of intensities for 10^1^ and 9^1^, presumably again due to the nature of the “Freq”-type hybrid scheme.

Switching to the 1150–1350 cm^–1^ region, the strong experimental peak at 1187 cm^–1^ ^67^ is assigned to 13^1^ (88%), which is calculated at 1181.1 cm^–1^ with an intensity of 108.8 km/mol. It is the most intense band in the whole 300–1600 cm^–1^ region according to these and previous computations (1179.9 cm^–1^, 98.8 km/mol),^64^ in line with experiment. Several weak bands are also reported in the 1190–1315 cm^–1^ range, at 1192, 1217, 1283, 1304, and 1313 cm^–1^, respectively. They are all successfully reproduced by our calculations at 1199.4, 1203.0, 1263.0, 1299.2, and 1308.0 cm^–1^, respectively, with intensities of 1 to 5 km/mol, and assigned to combination bands. Among them, the band observed at 1217 cm^–1^ (w)^62,63^ is assigned to the 27^1^26^1^ combination predicted at 1203.0 cm^–1^, showing a larger intensity (3.4 km/mol) than the fundamental transition 12^1^ at 1216.4 cm^–1^ (0.1 km/mol). This is different from the result of Krasnoshchekov et al., where 12^1^ was predicted at 1214.4 cm^–1^ with an intensity of 2 km/mol.

The following part of the spectrum, within 850–1150 cm^–1^, is characterized by rather weak, similar-intensity transitions and significant resonances. Hence, the traditional assumptions to assign bands to the fundamental transitions, overtones, and combinations are again challenged. Thanks to anharmonic computations, the pattern can be reproduced, and bands assigned. The weak bands observed at 1066 and 1073 cm^–1^ ^62^ are well reproduced at 1065.4 and 1073.3 cm^–1^, with intensities of 3.5 and 6.7 km/mol, respectively. They both have contributions from fundamental 14^1^, as well as from 20^1^18^1^ combination and 19^2^ overtone, in line with 1060.8 cm^–1^ (6.7 km/mol) by Krasnoshchekov et al.^64^ assigned as 14^1^ in resonance with 19^2^. Another medium peak at 1082 cm^–1^ ^67^ is assigned to 27^2^ (89%), which is predicted at 1090.1 cm^–1^ (9.1 km/mol). At lower energies, observed bands at 944 cm^–1^ ^101^ and 952 cm^–1^ ^67^ are assigned to 28^1^27^1^ (90%) calculated at 935.0 cm^–1^ with intensity of 6.6 km/mol, and 16^1^ (93%) at 954.3 cm^–1^ (9.9 km/mol). Another fundamental transition, 22^1^ (94%) is predicted at 958.9 cm^–1^ with a low intensity of 0.5 km/mol, and related to a weak experimental peak at 958 cm^–1^ (w), which is a shoulder of the band at 952 cm^–1^. This is in line with the assignments to the fundamental bands by Krasnoshchekov et al., at 947.5 cm^–1^ (10.4 km/mol) and 955.9 cm^–1^ (0.45 km/mol) for 16^1^ and 22^1^, respectively. Another fundamental transition, 15^1^ is in strong resonance with 29^1^23^1^, leading to two weak transitions at 973.6 cm^–1^ (2.0 km/mol) and 981.5 cm^–1^ (4.5 km/mol), in line with the experimental bands in 972 ^67^ and 984.7 cm^–1^.^63^ The assignment for 15^1^ is in line with the CVPT2//CC/MP2 study,^64^ which was predicted at 979.9 cm^–1^ (4.6 km/mol).

The most intense bands observed in the 550–850 cm^–1^ range can be assigned to essentially resonance-free fundamental transitions, 23^1^, 24^1^ and 26^1^ predicted at 810.1 (54.4 km/mol), 756.6 (30.6) and 659.4 cm^–1^ (72.2), which are consistent with the ones measured at 804,^62,63^ 761 (RS, s)^62^ and 662 cm^–1^ (s).^62^ Between these bands, we observe a few bands, related to resonance-free fundamental with medium intensity, 25^1^, (721.9 cm^–1^), and four weaker bands, two arising from 17^1^ in resonance with 18^2^, and two from combinations (30^1^19^1^ and 30^1^18^1^). The band at 757 cm^–1^(w) should be regarded as fundamental 17^1^ (84%), while another band at 792 cm^–1^ is related to overtone 28^2^ (85%), which gains intensity from 17^1^. Transitions to 30^1^19^1^ and 30^1^18^1^, predicted at 682.4 and 699.5 cm^–1^, respectively, are assigned to weak bands at 682 ^78^ and 692 cm^–1^ (vw).^67^ The proposed assignment for 23^1^ is in line with Krasnoshchekov et al., who calculated it at 803.2 cm^–1^ (51.6 km/mol) and associated it with the same experimental band. It should be noted that the reference experimental band chosen in this work (804 (s)^62^) is different from that of Krasnoshchekov et al. (802 (w)^67^). For 26^1^, 25^1^, 17^1^, and 24^1^, their predictions at 651.4 (32.4 km/mol), 715.8 (8.92), 751.8 (1.3), and 756.1 cm^–1^ (29.2), respectively, are consistent with our calculations, but the assignment to experimental bands does not match. For instance, the intense 26^1^ transition was linked to the weak band at 660 cm^–1^,^67^ and 17^1^ and 24^1^ are swapped with respect to our findings, likely due to the neglect of intensity redistributions.

In the spectral pattern between 500 and 550 cm^–1^, two fundamental transitions, 27^1^ and 20^1^, at 543.4 cm^–1^ (40.8 km/mol) and 512.8 cm^–1^ (18.3 km/mol) are not affected by resonances and related to the peaks measured at 551 cm^–1^ (m)^62^ and 512 cm^–1^ (w).^67^ The other two fundamental transitions, 18^1^ and 19^1^, are involved in Fermi interactions with 29^1^28^1^ and 30^1^28^1^, respectively, giving rise to the transitions computed at 532.9 (5.6 km/mol), 538.1 (1.1), and 549.8 cm^–1^ (4.4). These theoretical predictions are in qualitative agreement with the CVPT2//CC/MP2 results by Krasnoshchekov et al., but again, some differences in the assignment of the observed bands can be noted. In this work, among the experimental bands at 538, 545 cm^–1^(w) and 551 cm^–1^(m), the latter is assigned to 27^1^, while in ref 64, 27^1^ was predicted at 549.4 cm^–1^ (42.4 km/mol) and was thus assigned to the weak band at 545 cm^–1^.

The last group of weak bands observed in the IR spectrum, at 374 (vw) and 395 cm^–1^ (w),^67^ are related to fundamental transitions of similar intensity; 21^1^ and 28^1^, respectively, at 387.7 cm^–1^ (20.0 km/mol) and 391.5 cm^–1^ (22.1 km/mol). This assignment is opposite to ref 64, where both states were predicted at 385 ± 1 cm^–1^. Clearly, as both transitions are predicted with similar energies and intensities, they cannot be easily distinguished.

The two lowest-energy transitions, of A” symmetry, have not been directly observed in FTIR spectra, but their energies have been estimated from electronic fluorescence spectra in a supersonic jet^104^ to be at 205 and 149 cm^–1^. Additionally, neutron inelastic scattering (NIS) spectra of polycrystalline uracil samples^69,103^ measured energy levels at 216, 201, 166, 130, and 93. Following Krasnoshchekov et al.,^64^ we have connected the 30^1^ and 29^1^ transitions at 145.1 cm^–1^ and 164.3 cm^–1^ to fluorescence (149 cm^–1^) and NIS data (166 cm^–1^), respectively.

Overall, the computed GVPT2 spectra agree well with the experimental results below 1600 cm^–1^, predicting the correct intensity distribution. The average precision in energy is within 5 cm^–1^, with the largest deviations below 20 cm^–1^.

## Conclusions

This work aimed at the definition of an effective computational protocol for the simulation of reliable infrared spectra, focusing on the applicability of hybrid QM1/QM2 schemes, IR intensities, and the spectral patterns. To this end, we have chosen the challenging case of uracil, whose IR spectrum is characterized by several strong nonfundamental transitions. The recently introduced intensity-specific methodology to account automatically for anharmonic resonances was confirmed to be effective in the simulation of Infrared spectra, paving the way to black-box applications of computational protocols based on VPT2. Furthermore, three hybrid schemes have been studied based on the harmonic wavenumbers, PES, and both PES and PS computed at a higher level than the anharmonic correction. In line with previous studies,^33,42^ energy-based corrections considering only the wavenumbers or the PES led to an improvement of the prediction of transition energies with respect to pure lower-level calculations. However, corrections to both the PES and PS are needed to improve the overall accuracy of the simulated band shapes. Notably, spectral regions with strong anharmonic resonances are particularly sensitive to the definition of the hybrid model. Thus, to obtain correct IR intensities, the same level of care is necessary for the prediction of energy and property derivatives. This means using the same level of electronic structure theory for the whole harmonic and anharmonic components, ensuring their compatibility with normal-mode consistency checks.

The whole range of the IR spectrum of uracil was considered here, divided by regions, yielding overall a very good agreement with experimental results, providing also predictions in spectral regions not studied experimentally for isolated uracil up to NIR. For ranges dominated by strong, nonfundamental transitions (1600–1800 cm^–1^), some discrepancies in the band position can be attributed to the harmonic component of the potential energy, which, for the higher-level method employed in this work (revDSD-PBEP86-D3­(BJ)) indeed shows mean and maximum errors of about 6.1 and 17.1 cm^–1^ with respect to the best theoretical estimates. However, the latter, obtained by a composite scheme based on coupled-cluster computations,^33^ can only be used in the “Freq” hybrid scheme. This work highlights the need for coherent inclusion of energy and dipole moment parts for correct intensity distribution. Therefore, future developments in electronic structure QM methodologies that allow us to obtain all harmonic properties at higher accuracy are still very much sought.

Overall, a very accurate simulation of the IR spectra for uracil has been achieved by employing recent developments in second-order perturbation theory. All experimentally available spectra of uracil could be interpreted by our simulation, with bands not yet experimentally available also considered. These simulated data provide information for further higher-resolution studies of uracil in a wider range, including nonfundamental bands in the MIR and NIR ranges, also regions of 3-quanta transitions. The protocol and methods used in this work also provide important insights for other vibrational spectra studies, such as Raman, ROA, and VCD, where more properties are involved and even higher sensibility to the applied methodology is expected.

## Supplementary Material





## Notes

Structural parameters of the uracil optimized at the levels of revDSD-PBEP86-D3­(BJ) and B3PW91-D3­(BJ) in conjunction with jun-cc-pVTZ, compared to theoretical and semiexperimental references; analysis of the potential energy distribution for revDSD-PBEP86-D3­(BJ)/jun-cc-pVTZ; harmonic wavenumbers and intensities calculated by both DFTs, along with the best estimated ones; examples of input files for hybrid schemes “Freq”, “PES” and “PES+PS”; GVPT2 anharmonic frequencies and intensities of bands in the 1600–1800 cm^–1^ region by hybrid schemes “PES” and “Freq”; compilation of all relevant experimental data; theoretical spectra for different regions showing more detailed information for less intense bands; comparison of GVPT2 spectra with 2 quanta numbers and 3 quanta numbers for region 3600–7000 cm^–1^; and GVPT2 spectrum compared to IDVPT2 spectrum and also experimental spectra for the fingerprint region (PDF)
GVPT2 stick spectrum computed by the “PES+PS” hybrid scheme is provided (PES–PS_full_3Q_GVPT2.csv) (ZIP)
