# Supplementary material for: Reliable Modeling of Anharmonic Spectra Line-Shapes from VPT2 and Hybrid QM Models: IR Spectrum of Uracil as a Test Case
Source: J Phys Chem A. 2025 Jun 25;129(26):5860–80. doi: 10.1021/acs.jpca.5c02226 (PMC12235615; doi:10.1021/acs.jpca.5c02226)
Supplement: Supplementary file 1 [file jp5c02226_si_001.pdf]

Supporting Information: Reliable Modeling of Anharmonic Spectra Line-shapes from VPT2 and Hybrid QM Models: IR Spectrum of Uracil as a Test Case

Ruiqin Xu,† Qin Yang,*,‡ Julien Bloino,*,¶ and Malgorzata Biczysko*,§

†*Department of Physics, College of Sciences, Shanghai University, 99 Shangda Road, Shanghai, 200444, China*

‡*Institute of Organic Chemistry and Biochemistry, Czech Academy of Science, Flemingovo náměstí 2 16610 Prague, Czech Republic*

¶*Classe di Scienze, Scuola Normale Superiore, Piazza dei Cavalieri 7, 56126 Pisa, Italy*

§*Faculty of Chemistry, University of Wroclaw, F. Joliot-Curie 14, 50-383, Wroclaw, Poland*

E-mail: qin.yang@uochb.cas.cz; julien.bloino@sns.it; malgorzata.biczysko@uwr.edu.pl

List of Tables

S1 Equilibrium structure of uracil. Distances in Å, angles in degrees. 1

S2 (a) Harmonic wavenumbers (cm^{-1}) and intensities (km/mol) calculated with revDSD-PBEP86-D3(BJ)/jun-cc-pVTZ, along with the potential energy distributions for each vibrational mode. (b) Symmetry coordinate definitions for uracil. 2

S3 Harmonic wavenumbers (cm^{-1}) and intensities (km/mol) of best estimated,[1] revDSD-PBEP86-D3/jun-cc-pVTZ and B3PW91-D3/jun-cc-pVTZ 3

S4 Example of input file for hybrid scheme “Freq” 4

S5 Example of input file for hybrid scheme “PES” 4

S6 Example of input file for hybrid scheme “PES+PS” 4

S7 Calculated anharmonic frequencies and intensities of bands in the 1600–1800 cm^{-1} region from hybrid schemes “PES” and “Freq” 5

S8 Wavenumbers and intensities of observed bands. The values in bold are the most reliable ones. 6

List of Figures

S1 Theoretical spectra at the revDSD-PBEP86-D3(BJ)/B3PW91-D3(BJ) level (region below 1000 cm^{-1}). The simulated spectra are modeled with Lorentzian functions with half-widths at half-maximum = 1 cm^{-1} and grid step of 0.1 cm^{-1}. 8

S2 Theoretical spectra at the revDSD-PBEP86-D3(BJ)/B3PW91-D3(BJ) level (region 1000–1800 cm^{-1}). The simulated spectra are modeled with Lorentzian functions with half-widths at half-maximum = 1 cm^{-1} and grid step of 0.1 cm^{-1}. Most relevant bands are assigned, with the assignment for IDVPT2 shown as white on black. (a): subregion related to mode 5 to 8, (b): subregion related to mode 9 to 14. 10

S3 Theoretical spectra at the revDSD-PBEP86-D3(BJ)/B3PW91-D3(BJ) level (region 1800–2900 cm^{-1}). The simulated spectra are modeled with Lorentzian functions with half-widths at half-maximum = 1 cm^{-1} and grid step of 0.1 cm^{-1}. 11

S4 Theoretical spectra at the revDSD-PBEP86-D3(BJ)/B3PW91-D3(BJ) level (region 2900–3600 cm^{-1}). The simulated spectra are modeled with Lorentzian functions with half-widths at half-maximum = 1 cm^{-1} and grid step of 0.1 cm^{-1}. 12

S5 Theoretical spectra at the revDSD-PBEP86-D3(BJ)/B3PW91-D3(BJ) level (region 3600–7000 cm^{-1}). The simulated spectra are modeled with Lorentzian functions with half-widths at half-maximum = 1 cm^{-1} and grid step of 0.1 cm^{-1}. 13

S6 Theoretical spectra at the revDSD-PBEP86-D3(BJ)/B3PW91-D3(BJ) level (region 3600-7000 cm^{-1}) considering 2 quanta numbers and 3 quantum numbers respectively. The simulated spectra are modeled with Lorentzian functions with a half-width at half-maximum = 1 cm^{-1} and grid step of 0.1 cm^{-1}. 14

S7 Anharmonic GVPT2 and IDVPT2 spectra computed at the revDSD-PBEP86-D3(BJ)/B3PW91-D3(BJ) level with the jun-cc-pVTZ basis set, in the 1600-1800 cm^{-1} region, along with experimental spectra (Ivanov *et al.*[2] and Barnes *et al.*[3]). The simulated spectra are modeled with Lorentzian functions with half-widths at half-maximum = 1 cm^{-1} and grid step of 0.1 cm^{-1}. 15

Table S1: Equilibrium structure of uracil. Distances in Å, angles in degrees.

	revDSD-	B3PW91	r_e^{BO}			semi-GED	r_e^{SE}	
	PBEP86-D3(BJ)	-D3(BJ)	ref. 4	ref. 5	ref. 6	ref. 5	ref. 4	ref. 6
Distances								
N1–C2	1·3859	1·3848	1·3785	1·3796	1·3807	1·381(2)	1·3818(5)	1·3810(6)
C2–N3	1·3799	1·3746	1·3756	1·3775	1·3774	1·379(2)	1·3763	1·3749(7)
N3–C4	1·4040	1·4022	1·3974	1·4005	1·3993	1·402(2)	1·3979(4)	1·3991(6)
C4–C5	1·4571	1·4509	1·4539	1·4578	1·4558	1·454(8)	1·4550(6)	1·4548(6)
C5–C6	1·3466	1·3425	1·3433	1·3446	1·3435	1·339(18)	1·3450(6)	1·3429(7)
C6–N1	1·3733	1·3655	1·3723	1·3729	1·3726	1·374(2)	1·3720(6)	1·3722(7)
C2–O7	1·2141	1·2100	1·2112	1·2111	1·2099	1·210(1)	1·2103(2)	1·2101(4)
C4–O8	1·2169	1·2126	1·2138	1·2131	1·2126	1·212(1)	1·2128(2)	1·2186(4)
N1–H9	1·0067	1·0058	1·0046	1·0049	1·0045	1·005(10)	1·0043(12)	––
N3–H10	1·0109	1·0096	1·0090	1·0090	1·0088	1·009(10)	1·0083(12)	––
C5–H11	1·0783	1·0776	1·0766	1·0764	1·0761	1·076(13)	1·0757(12)	––
C6–H12	1·0818	1·0816	1·0793	1·0794	1·0792	1·079(13)	––	1·0781(12)
	revDSD-	MAE	0·0033	0·0025	0·0029	0·0030	0·0031	0·0035
	PBEP86-D3(BJ)	|MAX|	0·0074	0·0063	0·0051	0·0076	0·0061	0·0050
	B3PW91	MAE	0·0025	0·0027	0·0024	0·0025	0·0025	0·0031
	-D3(BJ)	|MAX|	0·0063	0·0074	0·0071	0·0085	0·0065	0·0067
Angles								
C2–N1–C6	123·46	123·53	123·38	123·60	123·45	123·8(3)	123·37(2)	123·39(4)
N1–C6–C5	121·99	122·00	121·91	121·69	121·93	121·6(4)	121·92(1)	121·90(2)
C6–C5–C4	119·64	119·73	119·49	119·66	119·58	120·0(3)	119·52(2)	119·61(3)
C5–C4–N3	113·66	113·58	113·97	113·82	113·76	113·6(3)	113·86(2)	113·75(4)
C4–N3–C2	128·08	128·12	127·75	127·73	127·97	128·0(3)	127·942	127·97(4)
N3–C2–N1	113·18	113·05	113·51	113·51	113·32	113·0(2)	113·383	113·38(4)
N1–C2–O7	122·87	122·71	122·87	122·81	122·92	122·6(5)	––	122·78(5)
N3–C2–O7	123·96	124·24	123·62	123·68	123·76	124·4(5)	123·88(4)	123·84(5)
C5–C4–O8	126·00	126·19	125·83	125·77	125·85	126·5(6)	125·77(5)	125·84(4)
C2–N1–H9	115·29	115·14	115·22	115·11	115·25	115·2(6)	––	115·14(12)
C2–N3–H10	115·57	115·64	115·70	115·70	115·60	114·(7)	––	115·62(13)
C6–C5–H11	121·86	121·91	122·11	122·01	122	122·(0)	––	122·12(11)
N1–C6–H12	115·39	115·42	115·34	115·49	––	112·4(4)	––	115·39(6)
	revDSD-	MAE	0·18	0·19	0·09	0·57	0·14	0·11
	PBEP86-D3(BJ)	|MAX|	0·34	0·35	0·20	2·99	0·23	0·26
	B3PW91	MAE	0·25	0·22	0·18	0·50	0·25	0·16
	-D3(BJ)	|MAX|	0·62	0·56	0·48	3·02	0·42	0·40

$\boldsymbol{r_e^{BO}}$**:** equilibrium structure calculated with composite schemes; $\boldsymbol{r_e^{SE}}$**:** semi-experimental equilibrium structure; semi-GED: Gas-phase electro diffraction (GED) data supplemented by MW rotational constants.

Table S2: (a) Harmonic wavenumbers (cm^{-1}) and intensities (km/mol) calculated with revDSD-PBEP86-D3(BJ)/jun-cc-pVTZ, along with the potential energy distributions for each vibrational mode. (b) Symmetry coordinate definitions for uracil.

(a) PED analysis

symm.	mode[a]	mode[b]	freq.	int.	CONTRIBUTIONS .. COORD(>5%)						
A'	1	1	3660.9	110.3	$\nu N1H$(100%)						
A'	2	2	3612.3	70.4	$\nu N3H$(100%)						
A'	3	3	3264.5	1.4	$\nu C5H$(95%)						
A'	4	4	3223.5	2.3	$\nu C6H$(95%)						
A'	5	5	1807.1	687.2	$\nu C2O$(68%)	-$\nu C2C3$(7%)	-$\nu N1C2$(6%)				
A'	6	6	1773.6	680.6	$\nu C4O$(66%)	-$\nu C4C5$(10%)	δring2(6%)				
A'	7	7	1684.4	38.5	$\nu C5C6$(58%)	-$\delta C6H$(14%)	-$\nu N1C6$(9%)	-$\nu C4O$(7%)			
A'	8	8	1511.5	107.8	$\delta N1H$(31%)	-$\nu N1C6$(28%)	$\nu C2O$(8%)	-$\nu C4C5$(6%)	$\nu N1C2$(5%)		
A'	9	9	1428.8	72.0	$\delta N1H$(22%)	$\delta C6H$(14%)	-$\nu C4C5$(12%)	$\nu C2C3$(12%)	$\delta C2O$(7%)	-$\delta C4O$(5%)	-$\nu N1C2$(5%)
A'	10	10	1418.1	42.7	$\delta N3H$(26%)	-$\delta C6H$(15%)	-$\delta C5H$(13%)	-$\nu N1C2$(12%)	$\delta C2O$(9%)	$\nu C3C4$(8%)	
A'	11	11	1395.0	18.0	$\delta N3H$(38%)	-$\nu C2C3$(14%)	$\delta C6H$(9%)	$\delta C5H$(8%)	$\nu N1C2$(7%)	$\nu C5C6$(6%)	$\delta N1H$(6%)
A'	12	12	1243.3	5.4	$\delta C5H$(23%)	-$\nu C3C4$(21%)	$\nu C2C3$(11%)	-$\nu N1C2$(10%)	$\nu C4C5$(10%)	$\nu N1C6$(8%)	$\delta C4O$(7%)
A'	13	13	1212.0	116.6	$\delta C6H$(30%)	$\nu C2C3$(17%)	-$\delta N1H$(17%)	-$\nu C3C4$(9%)	-$\nu N1C6$(6%)	-$\delta C5H$(5%)	
A'	14	14	1093.1	6.2	$\nu N1C6$(32%)	-$\delta C5H$(27%)	$\nu C5C6$(12%)	-$\nu C3C4$(6%)	$\delta N1H$(6%)	-$\nu N1C2$(5%)	
A'	15	15	996.9	7.5	δring1(74%)	-$\nu N1C2$(8%)					
A'	16	17	975.4	10.5	$\nu N1C2$(25%)	-$\nu C4C5$(22%)	$\delta N3H$(12%)	-$\nu C3C4$(10%)	$\nu C2C3$(9%)	-$\delta C2O$(7%)	
A'	17	19	773.9	3.7	$\nu C4C5$(28%)	$\nu N1C2$(17%)	δring1(14%)	$\nu C3C4$(9%)	$\nu C2C3$(7%)	δring3(7%)	$\nu N1C6$(6%)
A'	18	23	560.5	3.8	δring3(33%)	-$\delta C2O$(22%)	-$\delta C4O$(19%)	$\nu C4C5$(6%)	-$\nu N1C6$(5%)		
A'	19	25	541.8	6.4	δring3(33%)	$\delta C4O$(20%)	$\delta C2O$(18%)	-$\nu C3C4$(10%)	δring2(6%)		
A'	20	26	519.0	19.8	δring2(69%)	-δring3(6%)	$\delta N3H$(6%)	-$\delta C4O$(5%)			
A'	21	28	387.9	21.5	$\delta C4O$(33%)	-$\delta C2O$(31%)	δring3(8%)	$\nu C3C4$(8%)	δring2(7%)	$\nu C2C3$(6%)	
A"	22	16	979.4	0.3	$\gamma C6H$(69%)	-$\gamma C5H$(23%)					
A"	23	18	822.7	57.4	$\gamma C5H$(47%)	-$\gamma C4O$(29%)	τring1(8%)	$\gamma C6H$(8%)	-τring2(6%)		
A"	24	20	766.7	32.6	$\gamma C2O$(47%)	-τring1(31%)	-$\gamma N3H$(9%)				
A"	25	21	734.8	12.0	$\gamma C4O$(33%)	$\gamma C6H$(22%)	-τring1(21%)	$\gamma C5H$(8%)	-$\gamma C2O$(5%)		
A"	26	22	683.2	77.9	$\gamma N3H$(88%)						
A"	27	24	556.1	40.6	$\gamma N1H$(83%)	-$\gamma C5H$(6%)	-$\gamma C4O$(5%)				
A"	28	27	394.6	24.1	τring3(32%)	$\gamma N1H$(18%)	$\gamma C5H$(16%)	τring1(16%)	τring2(9%)	$\gamma C4O$(7%)	
A"	29	29	162.8	0.4	τring2(55%)	$\gamma N1H$(27%)	-τring3(10%)				
A"	30	30	145.9	1.0	τring1(43%)	$\gamma N3H$(30%)	-τring3(21%)				

a: Normal modes in descending order, sorted by irreducible representation;

b: Normal modes in descending order not sorted by irreducible representation.

(b) Coordinate definitions

Coordinate	Definition	Coordinate	Definition	Coordinate	Definition
$\nu N1C2$	R12	$\delta C2O$	$(A172 - A372)/\sqrt{2}$	δring1	$(A612 - A132 + A243 - A354 + A465 - A516)/\sqrt{6}$
$\nu N1C6$	R16	$\gamma C2O$	γ_{7132}	δring2	$(2A621 - A132 - A243 + 2A354 - A465 - A516)/\sqrt{12}$
$\nu N1H$	R19	$\delta N3H$	$(A2,10,3 - A4,10,3)/\sqrt{2}$	δring3	$(A132 - A243 - A465 - A516)/2$
$\nu C2C3$	R23	$\gamma N3H$	$\gamma_{10,2,4,3}$	τring1	$(\tau_{6123} - \tau_{1234} + \tau_{2345} - \tau_{3456} - \tau_{4561} - \tau_{5612})/\sqrt{6}$
$\nu C2O$	R27	$\delta C4O$	$(A384 - A584)/\sqrt{2}$	τring2	$(\tau_{6123} - \tau_{2345} + \tau_{3456} - \tau_{5612})/2$
$\nu C3C4$	R34	$\gamma C4O$	γ_{8354}	τring3	$(-\tau_{6123} + 2\tau_{1234} - \tau_{2345} - \tau_{3456} + 2\tau_{4561} - \tau_{5612})/\sqrt{12}$
$\nu C3H$	R3,10	$\delta C5H$	$(A4,11,5 - A6,11,5)/\sqrt{2}$		
$\nu C4C5$	R45	$\gamma C5H$	$\gamma_{11,4,6,5}$		
$\nu C4O$	R48	$\delta C6H$	$(A5,12,6 - A1,12,6)/\sqrt{2}$		
$\nu C5C6$	R56	$\gamma C6H$	$\gamma_{12,5,1,6}$		
$\nu C5H$	R5,11	$\delta N1H$	$(A691 - A291)/\sqrt{2}$		
$\nu C6H$	R6,12	$\gamma N1H$	γ_{9621}		

Table S3: Harmonic wavenumbers (cm^{-1}) and intensities (km/mol) of best estimated,[1] revDSD-PBEP86-D3/jun-cc-pVTZ and B3PW91-D3/jun-cc-pVTZ

symm.	mode	CC best estimated		revDSD-PBEP86-D3(BJ)		B3PW91-D3(BJ)	
		ω_0	A_0	ω	A	ω	A
A’	1	3652.7	112.4	3660.9	110.3	3659.6	105.2
A’	2	3602.2	72.5	3612.3	70.4	3615.2	67.0
A’	3	3252.8	2.1	3264.5	1.4	3255.8	1.5
A’	4	3217.5	1.8	3223.5	2.3	3208.7	2.2
A’	5	1790	726.0	1807.1	687.2	1816.0	603.3
A’	6	1761.5	720.3	1773.6	680.6	1782.0	767.1
A’	7	1677.5	34.1	1684.4	38.6	1686.5	70.5
A’	8	1505.4	105.8	1511.5	107.8	1509.3	102.3
A’	9	1427.2	102.9	1428.8	72.0	1424.4	54.4
A’	10	1414	4.6	1418.1	42.8	1413.8	61.6
A’	11	1394	12.2	1395.0	18.0	1385.5	14.5
A’	12	1248.2	19.6	1243.3	5.4	1235.5	0.6
A’	13	1205.3	110.6	1212.0	116.6	1206.2	105.0
A’	14	1084.2	6.4	1093.1	6.2	1095.3	5.4
A’	15	995.4	8.3	996.9	7.5	996.7	7.0
A’	16	973.3	10.9	975.4	10.5	973.3	8.4
A’	17	772.8	4.1	773.9	3.7	778.7	3.1
A’	18	558.7	3.6	560.5	3.8	561.1	4.4
A’	19	540.8	6.6	541.8	6.4	544.1	5.8
A’	20	517.2	20.6	519.0	19.8	521.4	21.0
A’	21	387.4	22.5	387.9	21.5	387.8	21.6
A"	22	967.7	0.5	979.4	0.3	982.2	0.1
A"	23	813.6	50.0	822.7	57.4	826.0	60.9
A"	24	765.2	31.5	766.7	32.6	774.7	34.4
A"	25	727.6	12.2	734.8	12.0	734.6	16.6
A"	26	670.3	81.9	683.2	77.9	689.4	67.0
A"	27	545.3	40.2	556.1	40.6	575.6	40.8
A"	28	387.9	24.0	394.6	24.1	400.9	21.5
A"	29	159.1	0.7	162.8	0.4	168.1	0.0
A"	30	140.4	0.9	145.9	1.0	150.5	1.9
			MAE	6.1	6.8	9.0	13.4
			MAX	17.1	38.2	30.3	57.0
			MIN	-4.9	-39.7	-12.7	-122.6

Table S4: Example of input file for hybrid scheme “Freq”

```
%Oldchk=checkpoint filename with anharmonic data
#P freq=(readanharm,readFC) geom=allcheck

PT2Model=GVPT2
DataSrc=NMOrder=AscNoIrrep
DataAdd=Freq

145.8902
......
3660.9098
```

Table S5: Example of input file for hybrid scheme “PES”

```
%Oldchk=checkpoint filename with anharmonic data
#P freq=(readanharm,readFC) geom=allcheck

PT2Model=GVPT2
DataAdd=PESHarm=inchk1

checkpoint filename with higher-level data
```

Table S6: Example of input file for hybrid scheme “PES+PS”

```
%Oldchk=checkpoint filename with anharmonic data
#P freq=(readanharm,readFC) geom=allcheck

PT2Model=GVPT2
DataSrc=(Harm=inchk1)

checkpoint filename with higher-level data
```

Table S7: Calculated anharmonic frequencies and intensities of bands in the 1600–1800 cm^{-1} region from hybrid schemes "PES" and "Freq"

(a) "PES"

state	freq.	full	ele	mcn	Polyad description
193	1781.3	222.9	254.2	230.8	0.701 $\|21^1 9^1\rangle$ -0.630 $\|5^1\rangle$ -0.235 $\|21^1 10^1\rangle$
192	1777.1	220.8	217.5	228.5	0.708 $\|21^1 9^1\rangle$ +0.567 $\|5^1\rangle$ +0.323 $\|21^1 10^1\rangle$
190	1768.5	79.3	89.3	85.2	0.673 $\|21^1 10^1\rangle$ -0.638 $\|18^1 12^1\rangle$ -0.304 $\|6^1\rangle$
189	1766.8	0.7	1.8	2.4	$\|23^1 22^1\rangle$
188	1766.0	70.9	75.5	74.6	0.622 $\|21^1 10^1\rangle$ +0.534 $\|18^1 12^1\rangle$ -0.420 $\|5^1\rangle$ +0.285 $\|6^1\rangle$
186	1756.8	243.5	250.1	245.1	0.578 $\|19^1 12^1\rangle$ +0.558 $\|6^1\rangle$ -0.485 $\|18^1 12^1\rangle$
183	1745.2	1.9	1.3	1.9	0.943 $\|21^1 11^1\rangle$ -0.327 $\|19^1 12^1\rangle$
182	1741.5	58.9	52.4	61.7	0.571 $\|19^1 12^1\rangle$ +0.526 $\|17^1 15^1\rangle$ -0.481 $\|18^1 13^1\rangle$ -0.266 $\|6^1\rangle$ +0.244 $\|21^1 11^1\rangle$
181	1737.4	15.6	17.3	16.7	0.788 $\|17^1 15^1\rangle$ +0.501 $\|18^1 13^1\rangle$
179	1734.0	140.5	154.9	143.3	0.667 $\|18^1 13^1\rangle$ -0.429 $\|6^1\rangle$ +0.368 $\|19^1 12^1\rangle$ -0.292 $\|19^1 13^1\rangle$ +0.261 $\|17^1 16^1\rangle$
176	1721.6	10.1	11.0	9.1	0.941 $\|20^1 12^1\rangle$ +0.273 $\|19^1 13^1\rangle$
174	1717.2	21.4	19.5	21.8	0.821 $\|19^1 13^1\rangle$ +0.470 $\|17^1 16^1\rangle$ -0.222 $\|20^1 12^1\rangle$
171	1706.8	168	161.9	172.3	0.809 $\|17^1 16^1\rangle$ +0.436 $\|6^1\rangle$ -0.340 $\|19^1 13^1\rangle$
169	1695.6	9.4	10.9	9.8	0.989 $\|20^1 13^1\rangle$
166	1650.5	75.5	61.9	78.3	0.953 $\|7^1\rangle$

(b) "Freq"

state	freq.	full	ele	mcn	Polyad description
193	1781.2	208.5	238.5	215.7	0.724 $\|21^1 9^1\rangle$ -0.615 $\|5^1\rangle$ -0.231 $\|21^1 10^1\rangle$
192	1776.9	233.7	231.8	241.6	0.685 $\|21^1 9^1\rangle$ +0.596 $\|5^1\rangle$ +0.345 $\|21^1 10^1\rangle$
190	1767.5	95.0	109.3	101.8	0.870 $\|21^1 10^1\rangle$ -0.302 $\|5^1\rangle$ -0.299 $\|18^1 12^1\rangle$
189	1766.8	0.7	1.8	2.4	$\|23^1 22^1\rangle$
188	1764.2	129.5	132.5	134.9	0.665 $\|18^1 12^1\rangle$ +0.504 $\|6^1\rangle$ +0.304 $\|19^1 12^1\rangle$ -0.281 $\|5^1\rangle$ +0.263 $\|21^1 10^1\rangle$
185	1755.2	173.3	178.4	173.2	0.641 $\|18^1 12^1\rangle$ -0.549 $\|19^1 12^1\rangle$ -0.450 $\|6^1\rangle$
183	1745.2	1.8	1.3	1.7	0.947 $\|21^1 11^1\rangle$ -0.318 $\|19^1 12^1\rangle$
182	1740.8	83.2	78.0	86.3	0.649 $\|17^1 15^1\rangle$ +0.552 $\|19^1 12^1\rangle$ -0.293 $\|6^1\rangle$ -0.287 $\|18^1 13^1\rangle$ +0.225 $\|21^1 11^1\rangle$
181	1736.6	58.2	59.8	61.0	0.720 $\|17^1 15^1\rangle$ -0.369 $\|19^1 12^1\rangle$ +0.339 $\|6^1\rangle$ +0.308 $\|18^1 13^1\rangle$
177	1730.9	70.1	81.8	70.9	0.873 $\|18^1 13^1\rangle$ -0.276 $\|6^1\rangle$ -0.233 $\|19^1 13^1\rangle$ +0.221 $\|17^1 16^1\rangle$
176	1721.6	10.4	11.4	9.4	0.944 $\|20^1 12^1\rangle$ +0.259 $\|19^1 13^1\rangle$
174	1717.0	20.1	18.3	20.5	0.822 $\|19^1 13^1\rangle$ +0.478 $\|17^1 16^1\rangle$
171	1706.7	170	164.2	174.2	0.802 $\|17^1 16^1\rangle$ +0.438 $\|6^1\rangle$ -0.346 $\|19^1 13^1\rangle$
169	1695.4	9.4	10.9	9.7	0.989 $\|20^1 13^1\rangle$
166	1650.4	75.5	62.3	78.5	0.956 $\|7^1\rangle$

full, ele, mcn: IR intensities (in units of km/mol) considering full, only electrical and mechanical anharmonicities, respectively.

Table S8: Wavenumbers and intensities of observed bands. The values in bold are the most reliable ones.

Ivanov et al.[2] (Ar)		Maltese M. et al.[7] (Ar)		Barnes *et al.*[3] (Ar)		Szczesniak *et al.*[8] (Ar)		Colarusso *et al.*[9] (Gas)		Graindourze *et al.*[10] (Ar)	Lés A. *et al.*[11] (Ar)		Chin S. *et al.*[12] (Ar)	
freq.	int.	freq.	int.	freq.	int.	freq.	int.	freq.	int.	freq.	freq.	int(rel.)	freq.	int(rel.)
3484.3		3490	m	3485	s	3482	125	**3484**	s	3485	3485	194	3482	166
**3479**	69	3484	vvw								3480	sh		
3472.8		3477	vvw			3467	100				3474	sh		
3435.5	48	3440	m	3435	s	3433	100	**3436**	s	3435	3435	122	3433	100
						3422	75							
						3130	15	**3124**	m				3130	4
				**3084**	m									
						2970	15						2970	8
								1897	m					
1791.5		1790	vw								1792			
1774.3		1773	m	**1774**	s	1774	125				1775		1774	
**1770.2**		1769	m								1771			
1763.7	273	1763	vs	**1764**	vs			1756	vs					
**1762.8**						1762	672							680
1761.4		1761	vs	**1761**	vs					1764	1764		1762	
1757.5		1756	ms	**1757**	vs	1758	312			1762	1758		1758	
1741		1740	m	**1741**	s						1742			
1733.2		1733	m	**1733**	s						1733		1733	
1731.3	163	1730	ms	**1731**	s							1632		
1730.3						1733	350							
1728.2		1727	s	**1728**	vs						1730			
1717.3		1717	m	**1718**	m	1720	62				1719		1720	
1706.4		1706	s	**1706**	vs	1707	375	1703	(sh) vs	1706	1706		1707	
1705.3	137													291
1703.5		1703	sh											
1698.4		1698	m	**1698**	ms	1699	163				1699		1699	
		1683	w								1687			
		1678	w								1681			
1644	17	1643	m	1643	m	1644	100	**1641**	s	1643	1644	60	1644	33
											1609	4		
		1540	w											
1517	6	1519	w	1515	w	1525	25	**1515**	m		1517	15		
1474.2						1473	125							
1471.9		1471	m	1472	ms			**1461**	s	1472	1472		1473	83
1467.6	44										1466	119		
1465.0		1465	mw											
**1458.5**		1458	w			1461	25				1459		1461	7
											1425	6		
											**1422**			
1399.6	31	1400	m	1399	vs	1401	138	**1400**	s	1400	1399	79	1401	56
1396.7		1388	mw	1389	m	1389	38	**1387**	s	1389	1389	31	1389	21
				**1386**	mw						1387			

Continued on next page

Ivanov		Maltese M.		Barnes		Szczesniak		Colarusso		Graindourze	Lés A.		Chin S.	
et al.[2] (Ar)		et al.[7] (Ar)		*et al.*[3] (Ar)		*et al.*[8] (Ar)		*et al.*[9] (Gas)		*et al.*[10] (Ar)	*et al.*[11] (Ar)		*et al.*[12] (Ar)	
freq.	int.	freq.	int.	freq.	int.	freq.	int.	freq.	int.	freq.	freq.	int(rel.)	freq.	int(rel.)
											1380	9		
						1366	13	1371	(sh) m					
1359.3	1.5	1360	vw	1360	vw	1361	13	**1356**	(sh) m	1359	1359	7		
1313.3		1317	vw	**1313**	vw	1314	10				1314			
1306.2	7	1306	vw	**1304**	vw						1307	26		
1302.8											1303			
						**1283**	25							
1217.4	1	1216	vw	**1217**	w	1219	13			1217	1217	9	1219	4
		**1192**	vw							1192	sh			
1185		1185	m	1184	vs	1186	413	**1187**	s	1185	1185	144	1186	109
1184.4	58													
1183.5								1172	s		1176	sh		
1102.4	5	1103		1100	w			**1082**	m		1102	12		
1075.5	3	1073	w	**1073**	w	1076	50			1075	1076		1076	14
1068.7	3	1070	w	**1066**	w						1069	11		
											1065			
987.5	1.5	970	vw	982	sh			990	(sh) w	987	987	11		
981.5	0.7	960	vw			963	13	**972**	(sh) w	980	982		963	2
958.3	4	958	sh	**958**	w	958	25			958	958	11	958	7
								**952**	w					
											**944**	2		
				842	vw									
804.0	22	806	m	**804**	s	806	175	802	w	804	804	69		
											**792**	2		
759.2	1			RS **761**	s					759	759	5		
756.5	12	759	m	757	s	769	125	**757**	w	757	757	39		
717.4	3	720	w	718	w	719	14	**717**	vw	718	717	13	719	12
						685	14	**692**	vw					
						**682**	13							
662.1														
659.5	33	665	m	**662**	s	664	100	660	w	662	662	116		
657.3		660	sh											
		587	vw			585	13							
559		558	w	559	w	557	25			562	559	7	557	17
556		548	w	555	w	551	25				555			
551.2	24	554	m	**551**	m					551	550	63		
								**545**	w					
536.4	4	539	mw	536	w	537	25			537	536	9	537	7
516.5	12	520	m	516	m	516	125	**512**	w	516	516	38	516	23
		514	sh								514	sh		
		395	m	391	m	393	25	**395**	w		393(sh)	548	393	33
								**374**	vw		391			

For matrices, only argon matrix data are collected in this table;

Additional experimental data: **984.7** Ne. matrix, Ivanov *et al.*;[2] **538**, **528** Aamouche *et al.*;[13] **166**;[13,14] **149** UV.[15]

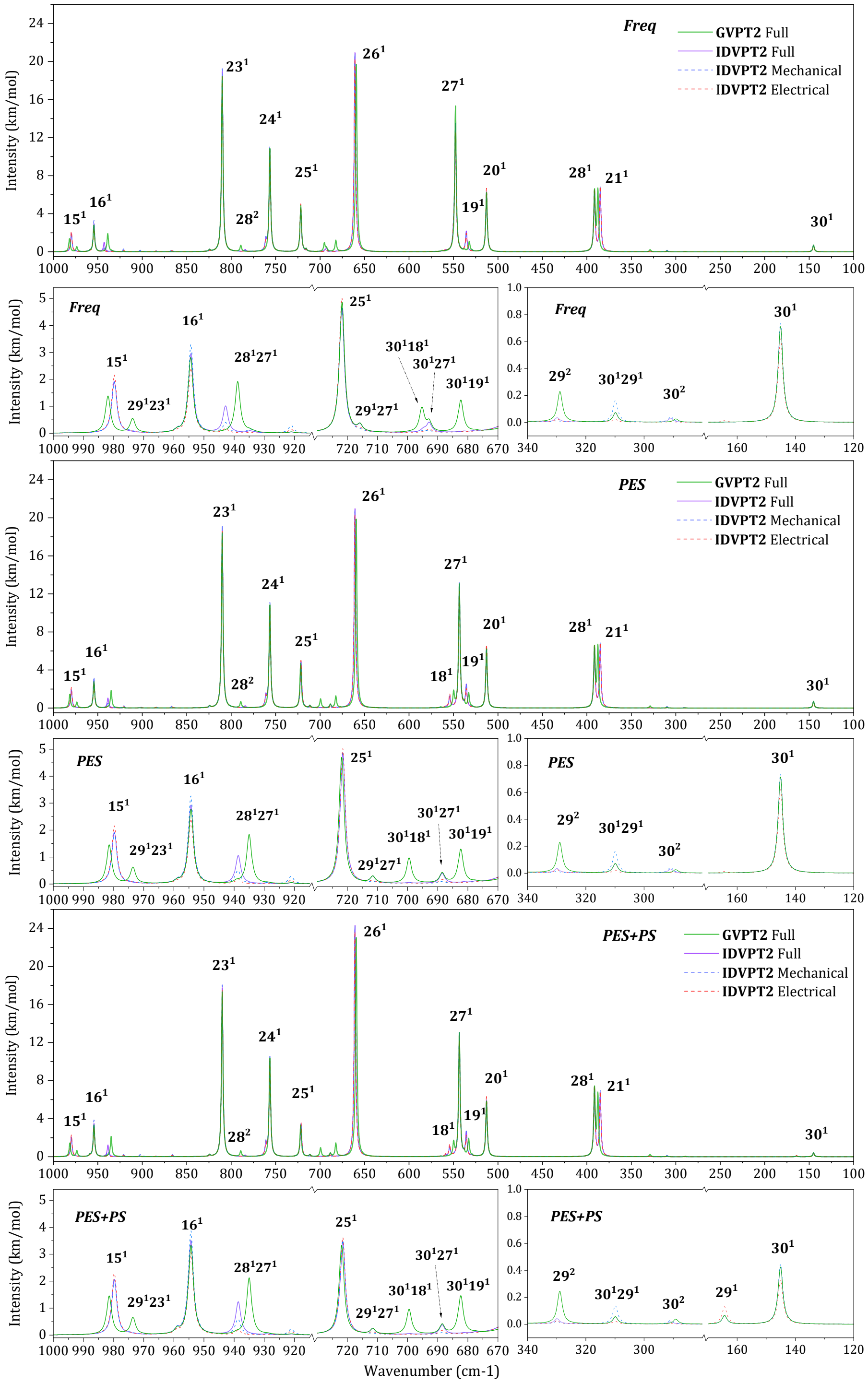

Figure S1: Theoretical spectra at the revDSD-PBEP86-D3(BJ)/B3PW91-D3(BJ) level (region below 1000 cm^{-1}). The simulated spectra are modeled with Lorentzian functions with half-widths at half-maximum = 1 cm^{-1} and grid step of 0.1 cm^{-1}.

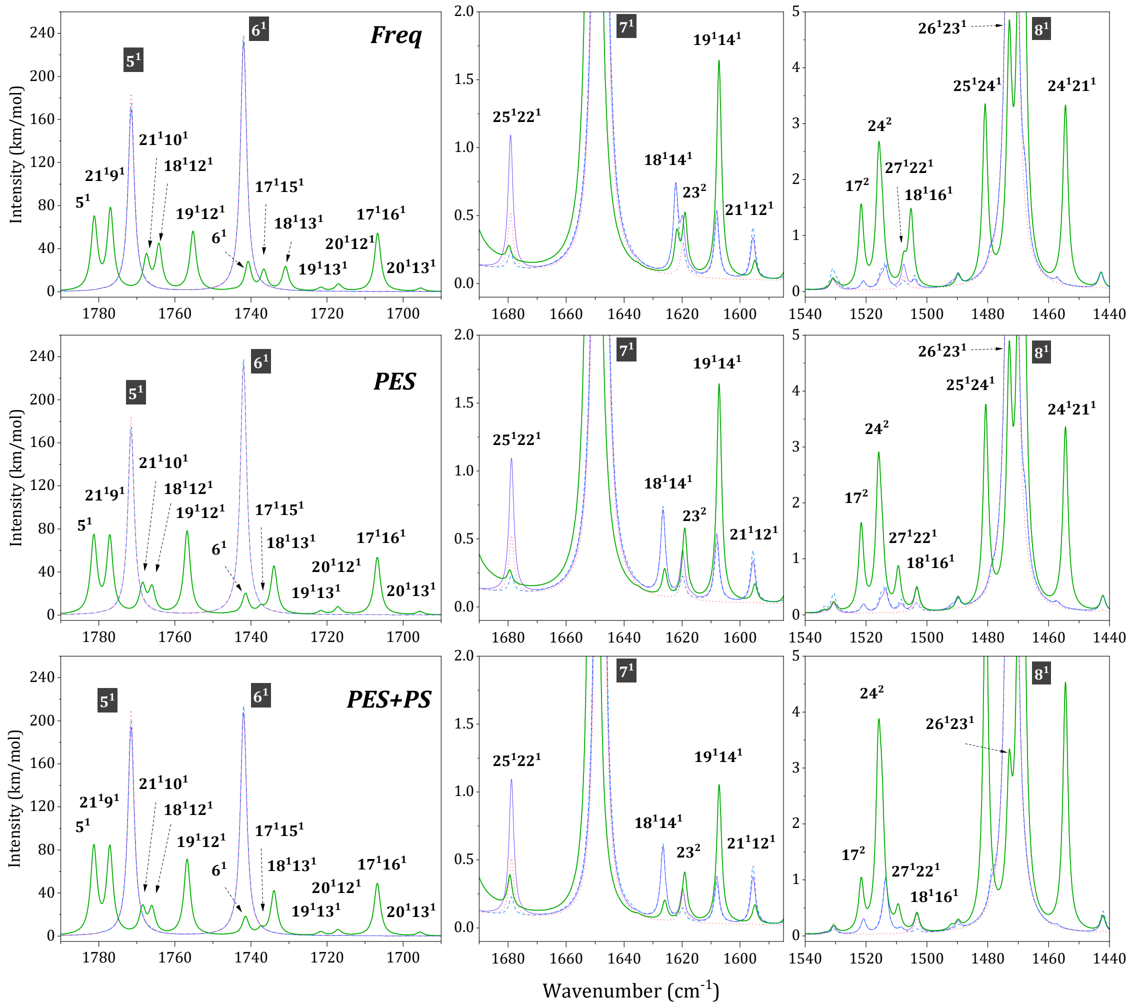

(a) subregion of $\nu5$ - $\nu8$

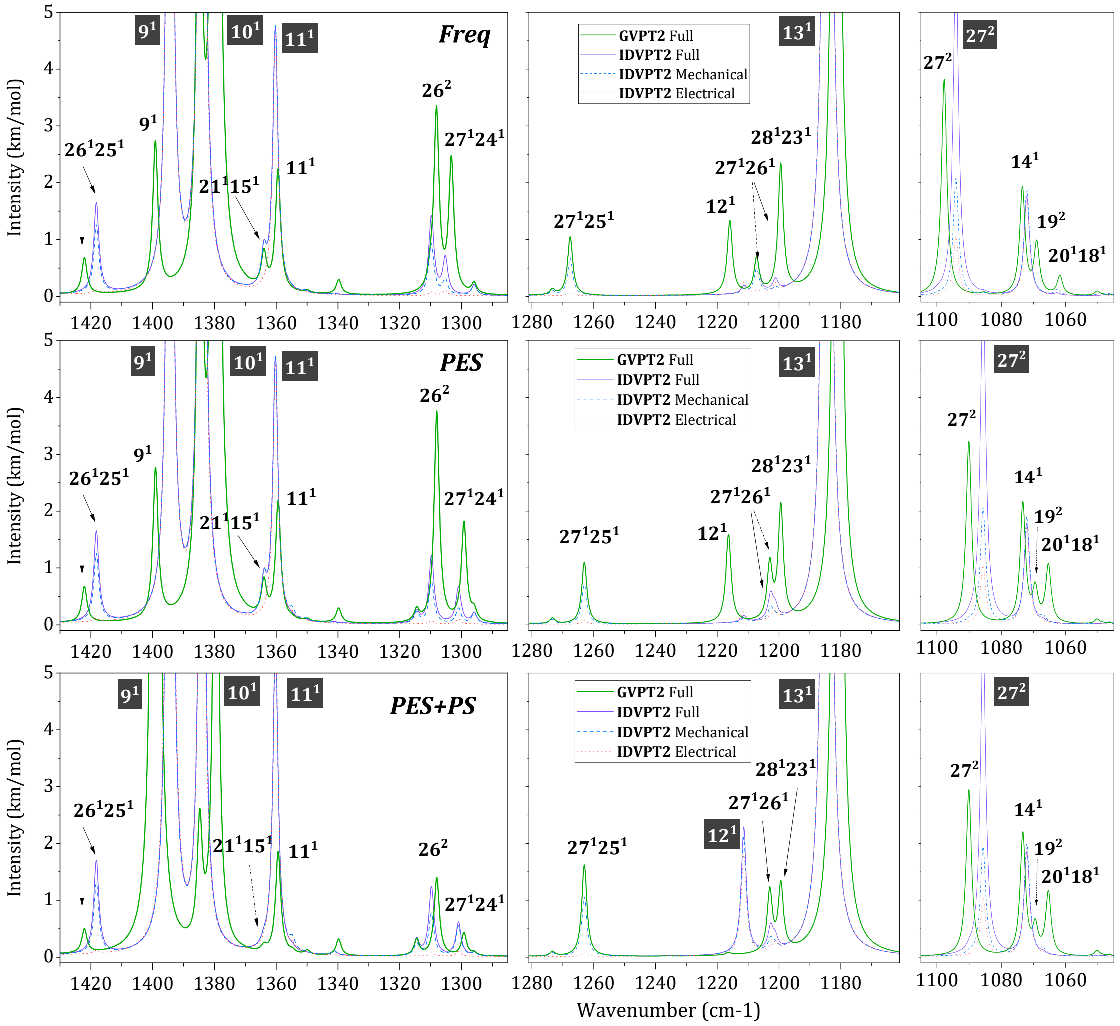

(b) subregion of $\nu 9$ - $\nu 14$

Figure S2: Theoretical spectra at the revDSD-PBEP86-D3(BJ)/B3PW91-D3(BJ) level (region 1000–1800 cm^{-1}). The simulated spectra are modeled with Lorentzian functions with half-widths at half-maximum = 1 cm^{-1} and grid step of 0.1 cm^{-1}. Most relevant bands are assigned, with the assignment for IDVPT2 shown as white on black. (a): subregion related to mode 5 to 8, (b): subregion related to mode 9 to 14.

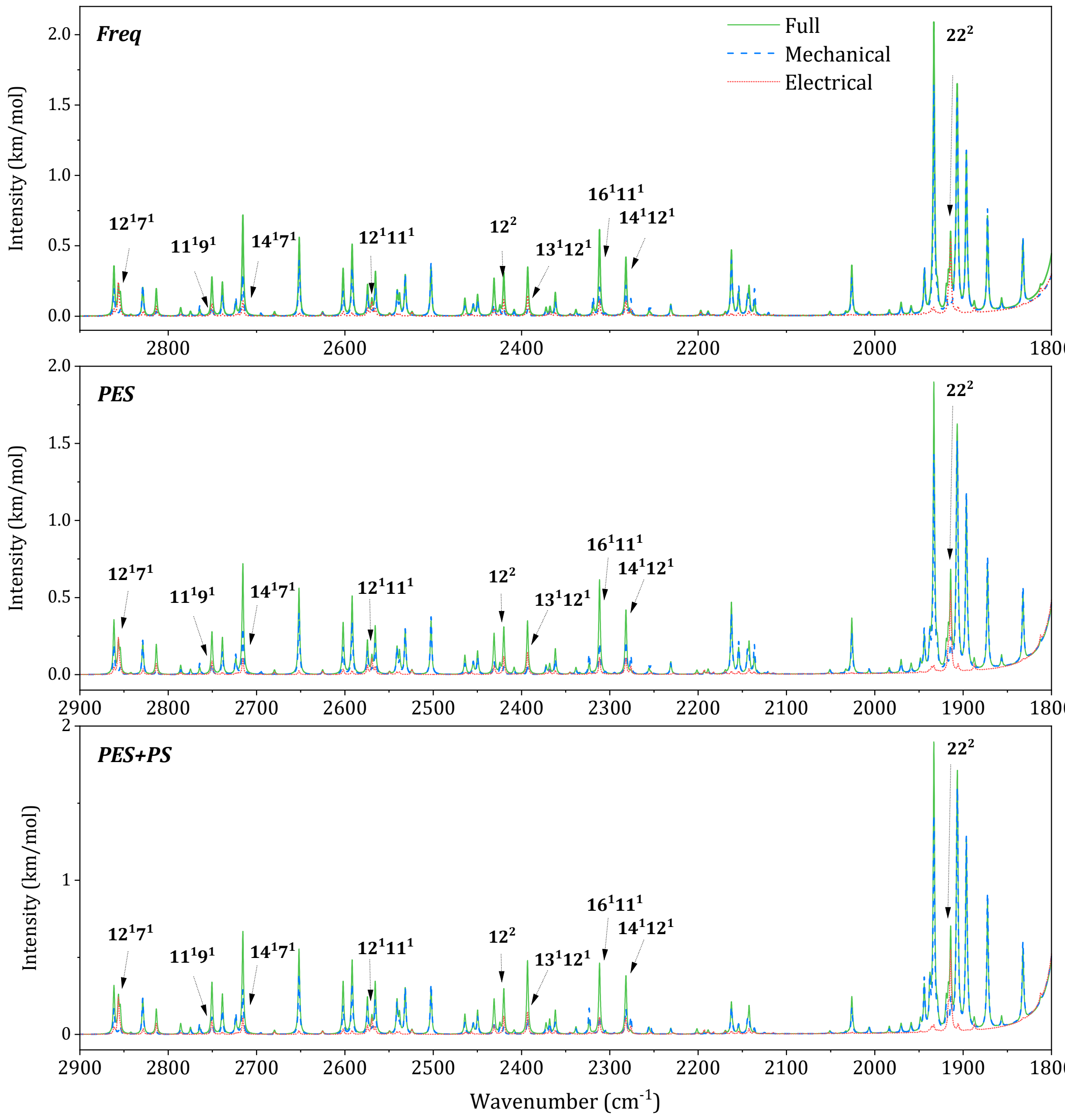

Figure S3: Theoretical spectra at the revDSD-PBEP86-D3(BJ)/B3PW91-D3(BJ) level (region 1800–2900 cm^{-1}). The simulated spectra are modeled with Lorentzian functions with half-widths at half-maximum = 1 cm^{-1} and grid step of 0.1 cm^{-1}.

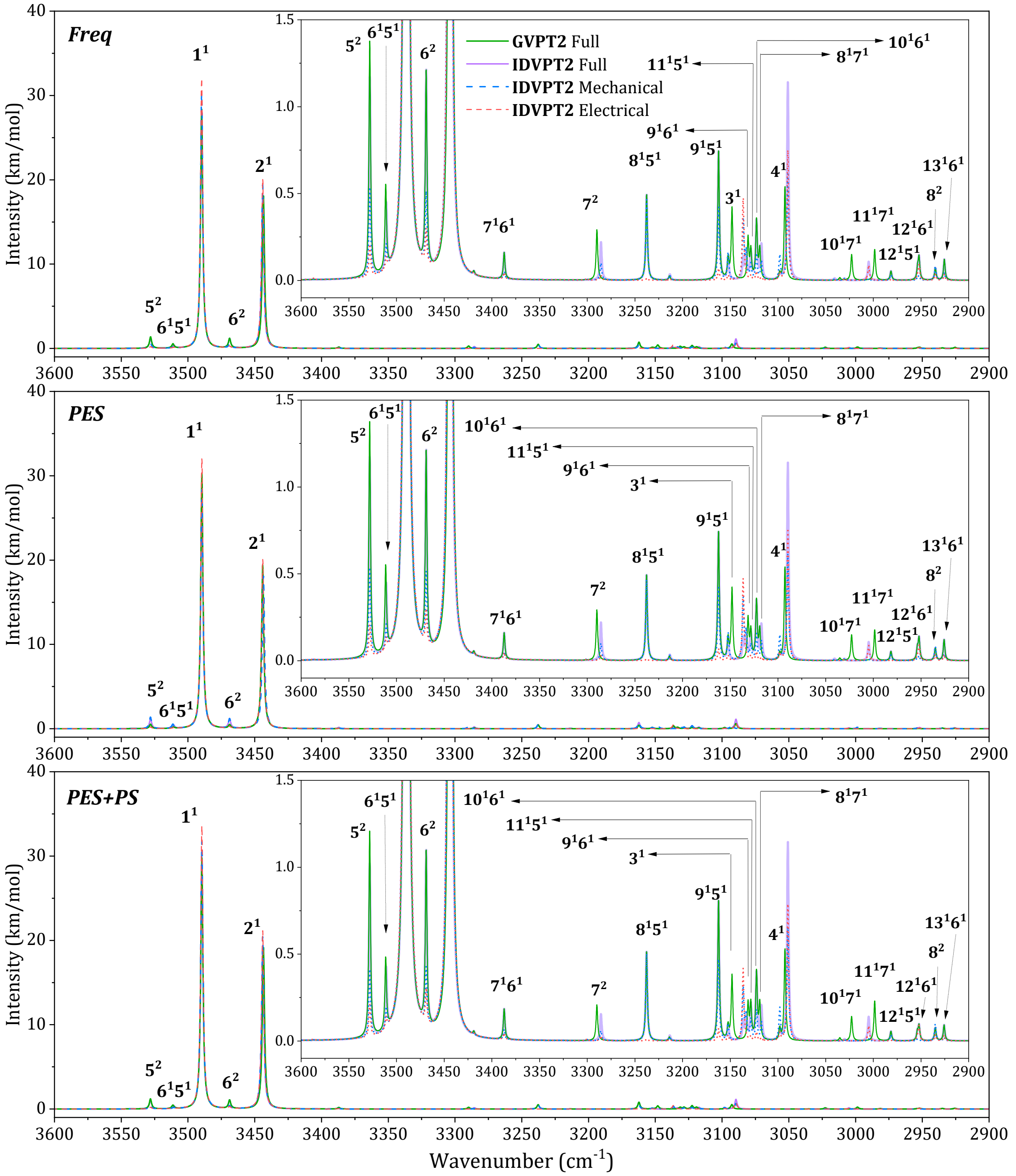

Figure S4: Theoretical spectra at the revDSD-PBEP86-D3(BJ)/B3PW91-D3(BJ) level (region 2900–3600 cm^{-1}). The simulated spectra are modeled with Lorentzian functions with half-widths at half-maximum = 1 cm^{-1} and grid step of 0.1 cm^{-1}.

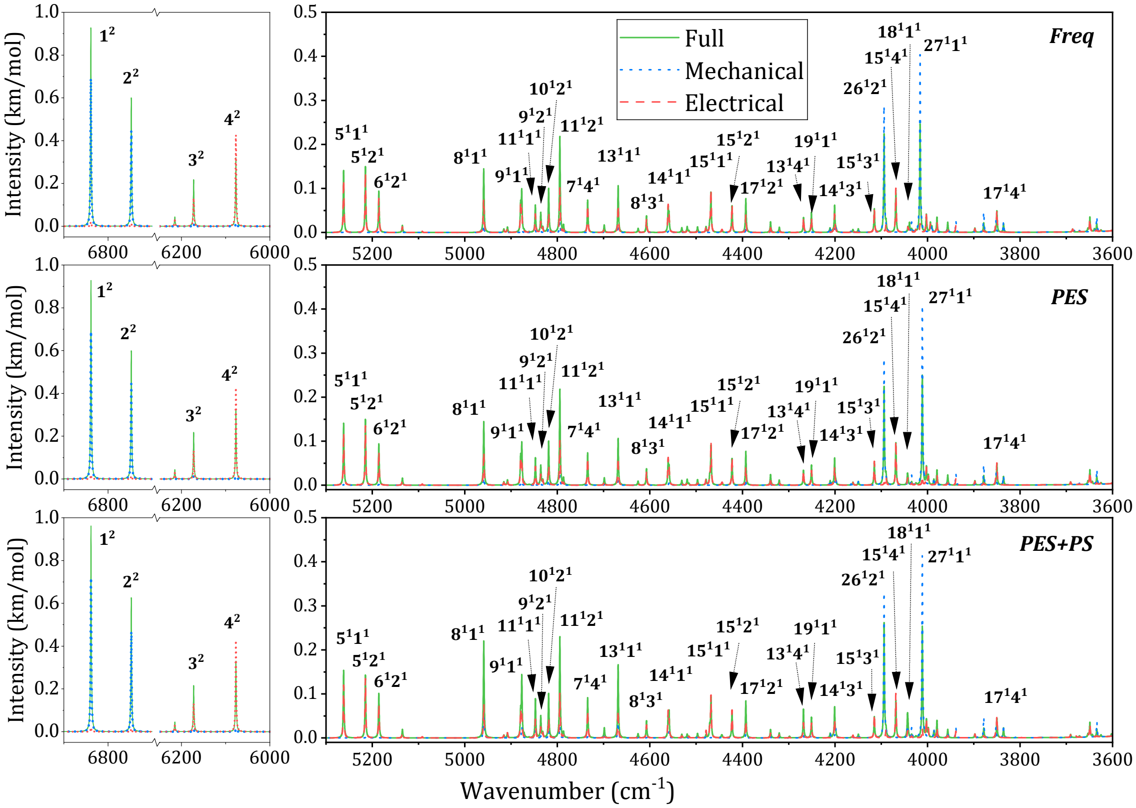

Figure S5: Theoretical spectra at the revDSD-PBEP86-D3(BJ)/B3PW91-D3(BJ) level (region 3600–7000 cm^{-1}). The simulated spectra are modeled with Lorentzian functions with half-widths at half-maximum = 1 cm^{-1} and grid step of 0.1 cm^{-1}.

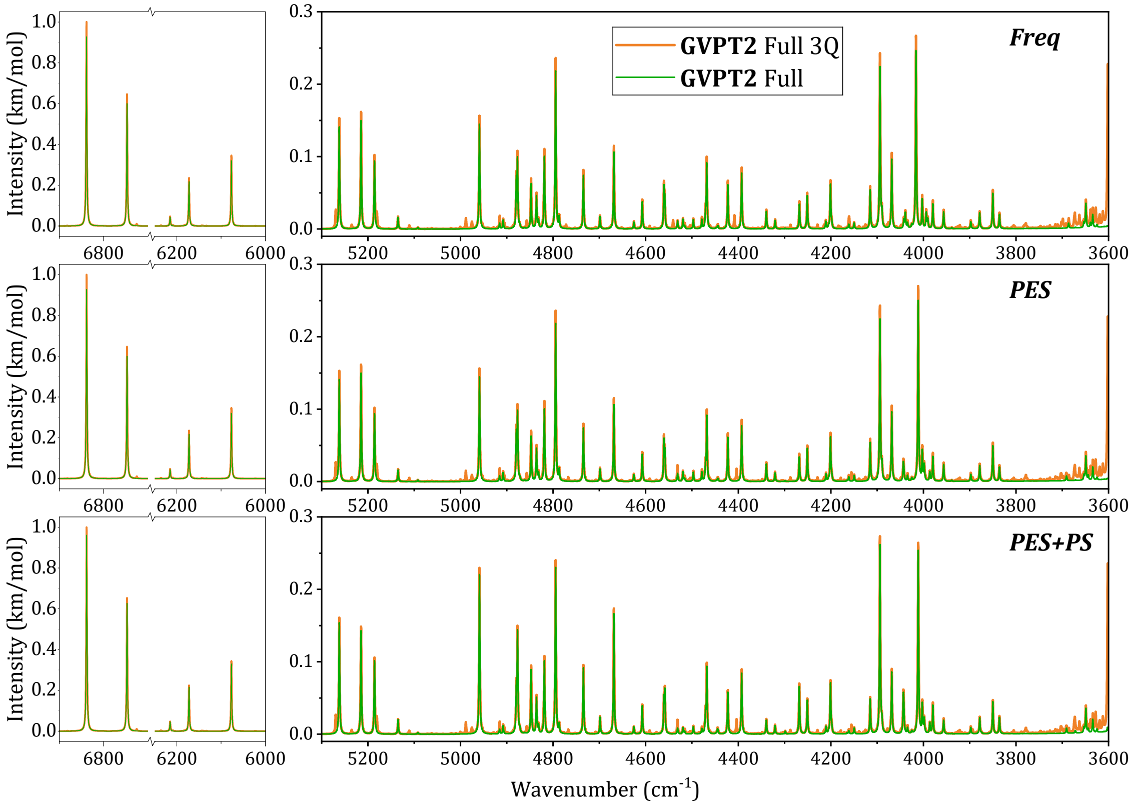

Figure S6: Theoretical spectra at the revDSD-PBEP86-D3(BJ)/B3PW91-D3(BJ) level (region 3600-7000 cm^{-1}) considering 2 quanta numbers and 3 quantum numbers respectively. The simulated spectra are modeled with Lorentzian functions with a half-width at half-maximum = 1 cm^{-1} and grid step of 0.1 cm^{-1}.

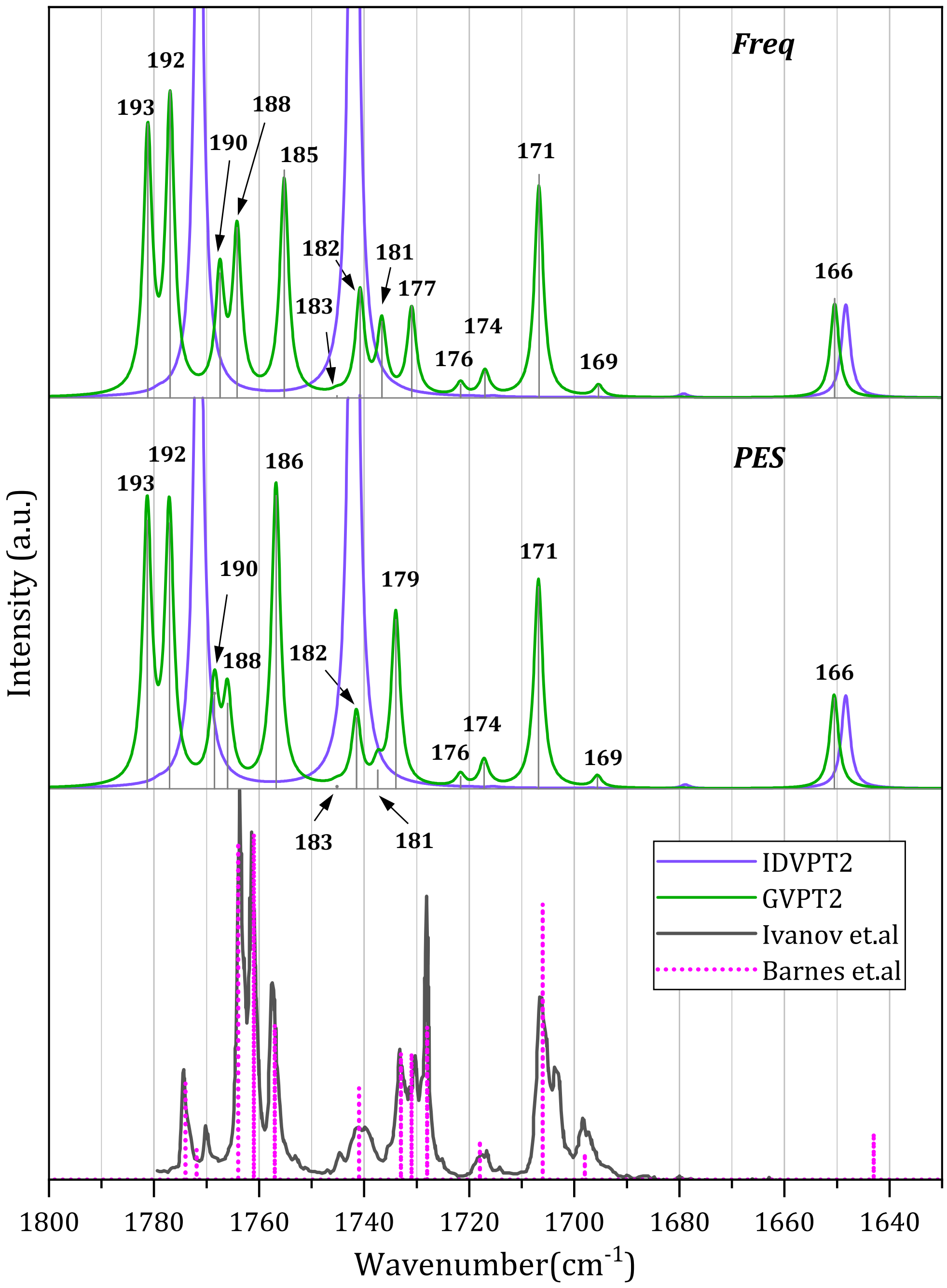

Figure S7: Anharmonic GVPT2 and IDVPT2 spectra computed at the revDSD-PBEP86-D3(BJ)/B3PW91-D3(BJ) level with the jun-cc-pVTZ basis set, in the 1600-1800 cm^{-1} region, along with experimental spectra (Ivanov *et al.*[2] and Barnes *et al.*[3]). The simulated spectra are modeled with Lorentzian functions with half-widths at half-maximum = 1 cm^{-1} and grid step of 0.1 cm^{-1}.

References

(1) Puzzarini, C.; Biczysko, M.; Barone, V. Accurate Anharmonic Vibrational Frequencies for Uracil: The Performance of Composite Schemes and Hybrid CC/DFT Model. *Journal of Chemical Theory and Computation* **2011**, *7*, 3702–3710.

(2) Ivanov, A.; Plokhotnichenko, A. M.; Radchenko, E. D.; Sheina, G. G.; Blagoi, Y. P. FTIR spectroscopy of uracil derivatives isolated in Kr, Ar and Ne matrices: matrix effect and Fermi resonance. *Journal of Molecular Structure: THEOCHEM* **1995**, *372*, 91–100.

(3) Barnes, A.; Stuckey, M.; Le Gall, L. Nucleic acid bases studied by matrix isolation vibrational spectroscopy: Uracil and deuterated uracils. *Spectrochimica Acta Part A: Molecular Spectroscopy* **1984**, *40*, 419–431.

(4) Puzzarini, C.; Barone, V. Extending the molecular size in accurate quantum-chemical calculations: the equilibrium structure and spectroscopic properties of uracil. *Phys. Chem. Chem. Phys.* **2011**, *13*, 7189–7197.

(5) Vogt, N.; Khaikin, L. S.; Grikina, O. E.; Rykov, A. N. A benchmark study of molecular structure by experimental and theoretical methods: Equilibrium structure of uracil from gas-phase electron diffraction data and coupled-cluster calculations. *Journal of Molecular Structure* **2013**, *1050*, 114–121.

(6) Császár, A. G.; Demaison, J.; Rudolph, H. D. Equilibrium Structures of Three-, Four-, Five-, Six-, and Seven-Membered Unsaturated N-Containing Heterocycles. *The Journal of Physical Chemistry A* **2015**, *119*, 1731–1746.

(7) Maltese, M.; Passerini, S.; Nunziante-Cesaro, S.; Dobos, S.; Harsànyi, L. Infrared levels of monomeric uracil in cryogenic matrices. *Journal of Molecular Structure* **1984**, *116*, 49–65.

(8) Szczesniak, M.; Nowak, M. J.; Rostkowska, H.; Szczepaniak, K.; Person, W. B.; Shugar, D. Matrix isolation studies of nucleic acid constituents. 1. Infrared

spectra of uracil monomers. *Journal of the American Chemical Society* **1983**, *105*, 5969–5976.

(9) Colarusso, P.; Zhang, K.; Guo, B.; Bernath, P. F. The infrared spectra of uracil, thymine, and adenine in the gas phase. *Chemical Physics Letters* **1997**, *269*, 39–48.

(10) Graindourze, M.; Smets, J.; Zeegers-Huyskens, T.; Maes, G. Fourier transform—infrared spectroscopic study of uracil derivatives and their hydrogen bonded complexes with proton donors: Part I. Monomer infrared absorptions of uracil and some methylated uracils in argon matrices. *Journal of Molecular Structure* **1990**, *222*, 345–364.

(11) Leś, A.; Adamowicz, L.; Nowak, M. J.; Lapinski, L. The infrared spectra of matrix isolated uracil and thymine: An assignment based on new theoretical calculations. *Spectrochimica Acta Part A: Molecular Spectroscopy* **1992**, *48*, 1385–1395.

(12) Chin, S.; Scott, I.; Szczepani, K.; Person, W. B. Matrix isolation studies of nucleic acid constituents. 2. Quantitative ab initio prediction of the infrared spectrum of in-plane modes of uracil. *Journal of the American Chemical Society* **1984**, *106*, 3415–3422.

(13) Aamouche, A.; Berthier, G.; Coulombeau, C.; Flament, J.; Ghomi, M.; Henriet, C.; Jobic, H.; Turpin, P. Molecular force fields of uracil and thymine, through neutron inelastic scattering experiments and scaled quantum mechanical calculations. *Chemical Physics* **1996**, *204*, 353–363.

(14) Aamouche, A.; Ghomi, M.; Coulombeau, C.; Jobic, H.; Grajcar, L.; Baron, M. H.; Baumruk, V.; Turpin, P. Y.; Henriet, C.; Berthier, G. Neutron Inelastic Scattering, Optical Spectroscopies and Scaled Quantum Mechanical Force Fields for Analyzing the Vibrational Dynamics of Pyrimidine Nucleic Acid Bases. 1. Uracil. *The Journal of Physical Chemistry* **1996**, *100*, 5224–5234.

(15) Fujii, M.; Tamura, T.; Mikami, N.; Ito, M. Electronic spectra of uracil in a supersonic jet. *Chemical Physics Letters* **1986**, *126*, 583–587.
